# Stimuli-responsive nanomaterials for wound healing: advances and emerging directions

**DOI:** 10.7150/thno.133095

**Published:** 2026-05-18

**Authors:** Yijia Dong, Yaqing Zhang, Junrong Wu, Yujun Yang, Yanli Zhang, Zhenjun Zhu, Rabiya Noor, Jawad Hussain, Longquan Shao, Yi Xing

**Affiliations:** 1 Stomatological Hospital, School of Stomatology, Southern Medical University, Guangzhou, 510280, PR China.; 2 Department of Stomatology, Shenzhen People’s Hospital, Shenzhen, 518020, PR China.; 3 Riphah International University, Lahore, Pakistan.; 4 Guangdong Provincial Key Laboratory of Construction and Detection in Tissue Engineering, Southern Medical University, Guangzhou, 510280, PR China.

**Keywords:** stimuli-responsive nanomaterials, wound healing, microenvironment, drug release, integrated diagnosis and treatment

## Abstract

In wound healing applications, conventional nanomaterials fail to precisely regulate the wound healing process because of the lack of dynamic adaptation to the wound microenvironment. Conversely, stimuli-responsive nanomaterials can respond to endogenous stimuli such as pH, enzymes, reactive oxygen species (ROS), and glucose, as well as exogenous stimuli such as light, electricity, and magnetism. Thus, stimuli-responsive nanomaterials can facilitate precise drug delivery and effectively treat wounds, and some of these materials can even achieve integrated diagnosis and treatment. Previous reviews have focused mainly on hydrogel carriers with stimuli-responsive properties. This review focuses on stimuli-responsive nanomaterials, classifies these materials on the basis of their stimulus sources, and systematically summarizes the response mechanisms, application outcomes, and design strategies of endogenous, exogenous, and dual stimuli-responsive nanomaterials applied in wound healing. Furthermore, this review explores the research gaps and future developments of stimuli-responsive nanomaterials for wound healing.

## 1. Introduction

Wound healing is among the most complicated processes in the human body and requires intricate spatial and temporal coordination of cells and tissues 1. However, due to the increasing prevalence of diseases such as diabetes, cardiovascular diseases, and autoimmune disorders, this process is often disrupted, resulting in chronic nonhealing wounds and even severe consequences such as amputation and death.

Nanomaterials and hydrogels are the most common strategies for wound treatment 2, 3. Nanomaterials refer to materials that have at least one dimension in the three-dimensional space within the nanoscale range (1–100 nm) or are composed of units of this scale as the basic building blocks 4. Hydrogels are three-dimensional network structures formed by natural or synthetic polymers through physical or chemical cross-linking 5. Because of their high specific surface area and small particle size, nanomaterials exhibit significant advantages over traditional hydrogels in promoting wound healing 6.

The large specific surface area of nanomaterials endows them with three major advantages: strong enzymatic catalytic activity, excellent drug-loading capacity, and the ability to regulate cellular biological functions. First, the large specific surface area of nanomaterials allows them to expose more active sites and increase the reaction area, enabling certain nanomaterials to have superior catalytic activity, achieving functions such as efficient sterilization, oxygen production, and catalytic decomposition of ROS, thereby regulating the microenvironment of the wound 7-10. In contrast, hydrogels usually need to be combined with nanomaterials with catalytic activity to achieve the corresponding effect 11. Second, the large specific surface area enables nanomaterials to achieve stable drug loading through various mechanisms, such as electrostatic adsorption, physical binding, and chemical bonding, thereby significantly enhancing drug-loading efficiency and accelerating wound healing through targeted drug delivery and controlled drug release 12. While most hydrogels rely primarily on physical encapsulation for drug delivery, if the interaction between the drug and the gel matrix is weak, stable and efficient drug loading is difficult to achieve, and drug burst release is likely to occur 13. Third, the high specific surface area of nanomaterials provides an important structural basis for regulating the biological behavior of cells. For example, the large specific surface area of nanofibers can provide a larger attachment area for skin tissue cells, effectively regulating cell proliferation and migration by mimicking the structure of the natural extracellular matrix, thereby accelerating wound healing 14.

In addition, the small particle size of nanomaterials allows them to better penetrate tissue and the bacterial biofilm barrier. Moreover, the size advantage also facilitates the arrival of nanomaterials at deep wound areas, thereby exerting corresponding biological effects 8. In bacteria-infected wounds, pathogenic bacteria aggregate and secrete extracellular polymers, forming a dense bacterial biofilm, which leads to persistent wound infection 15. With their small particle size, nanomaterials can effectively penetrate the dense extracellular matrix of the bacterial biofilm and kill bacteria deep within the biofilm, thereby eliminating wound infection 16. Moreover, nanomaterials can penetrate the dermis layer and even deeper damaged tissues through intercellular spaces or capillary walls to achieve deep drug delivery and promote deep wound healing 17. Traditional hydrogels usually cover only the wound surface and are difficult to penetrate the bacterial biofilm barrier or penetrate deep into the tissue to act on deep wounds 18.

**Figure 1:**
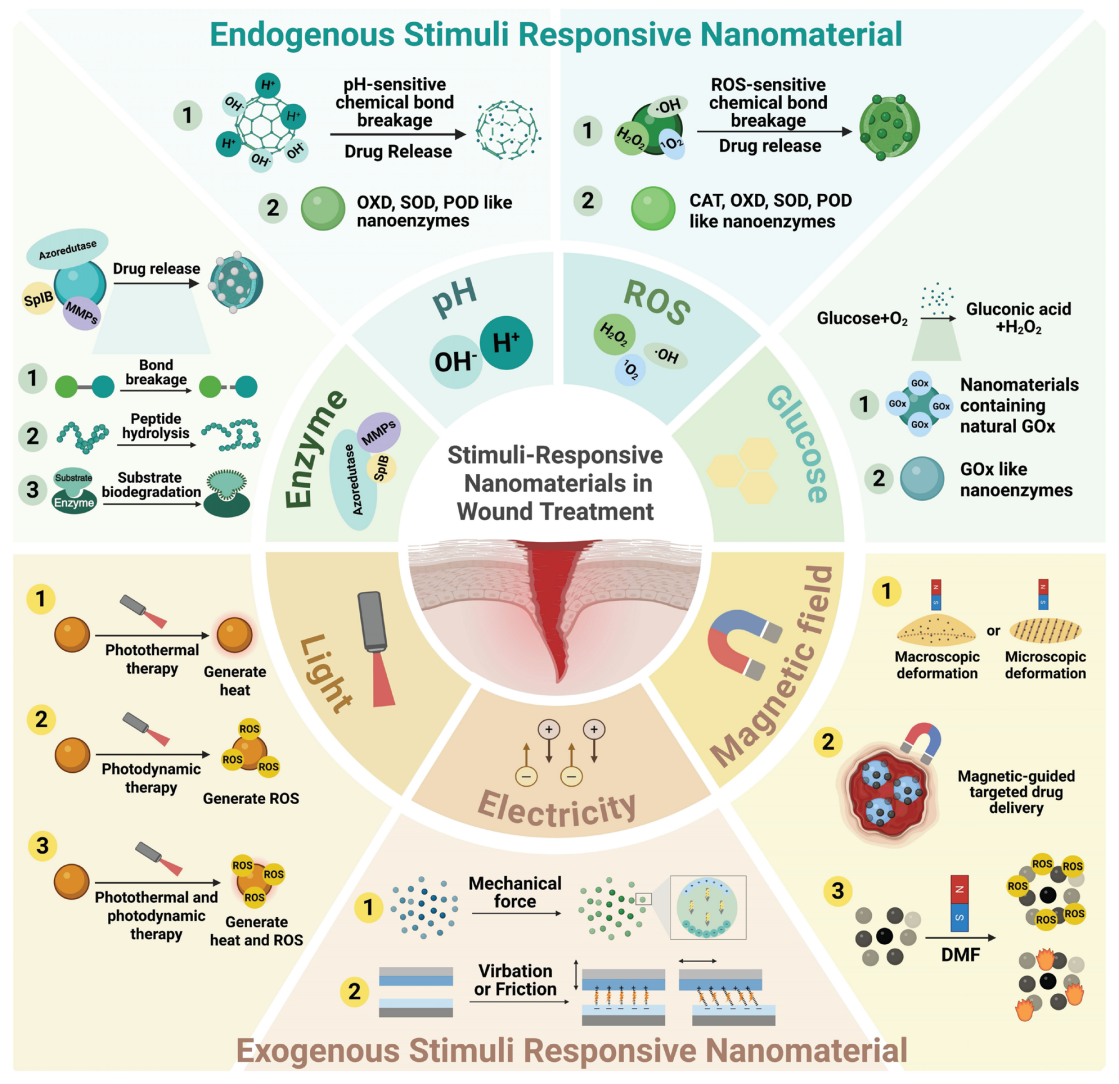
Schematic diagram illustrating the major categories of stimuli-responsive nanomaterials and their working mechanisms. The figure shows how nanomaterials are designed to respond to various endogenous stimuli and exgenous stimuli. Upon exposure to a specific trigger, the nanomaterials undergo energy conversion or structural change, leading to directed pro-healing effects or controlled release of therapeutic agents. Created in https://BioRender.com.

However, the complex pathological wound microenvironment, which is characterized by hyperglycemia, inflammation, infection, and hypoxia, results in abnormal physicochemical indicators, including pH, ROS, glucose, and enzyme expression profiles 19-21. Conventional nanomaterials cannot dynamically respond to such pathological microenvironments, and their effects are difficult to precisely control; therefore, they cannot achieve targeted intervention at the critical stage of wound healing, leading to problems such as off-target effects, pathogen resistance, and difficulty in adapting to complex wounds **(Figure 1)** 22,23. Therefore, stimuli-responsive nanomaterials have garnered increasing attention and research.

Stimuli-responsive nanomaterials refer to nanomaterials that can respond to different stimulus, thereby changing their physical and chemical structures or undergoing energy conversion 24. On the basis of the advantages of these nanomaterials, such as dynamically adapting to the wound microenvironment, preventing the development of drug resistance, targeted drug delivery, and promoting wound healing in multiple stages, new ideas and strategies for wound treatment have been introduced 25-28. To date, stimuli-responsive nanomaterials have demonstrated excellent antibacterial, anti-inflammatory, and immunomodulatory effects 29-32. These findings indicate the great application potential of these materials in skin wound treatment.

In the different types of wound microenvironments and at different stages of healing, endogenous indicators such as pH, enzymes, ROS, and glucose are constantly changing. For example, in bacteria-infected wounds, the expression levels of azide reductase and serine protease are relatively high 33,34, whereas in diabetic wounds, the expression levels of matrix metalloproteinases (MMPs) and local glucose concentrations are relatively high 35,36. Furthermore, certain indicators shift dynamically during different healing stages (including the hemostasis phase, inflammatory phase, proliferation phase and remodeling phase). For instance, the pH gradually increases from 5.5–6.0 during the inflammatory phase to 6.5–7.4 during the proliferation phase and the remodeling phase 37. Additionally, during the inflammatory phase, ROS levels abnormally increase but gradually decrease during the proliferative and remodeling periods, then remain at a relatively low level 38. Based on these features, researchers have designed nanomaterials that respond to endogenous stimulus. By accurately sensing microenvironmental cues (including pH, ROS, and enzymatic levels), these responsive materials can undergo programmed degradation or exhibit nanozyme-like activity, thereby synergistically promoting wound repair 39,40.

When exposed to external physical energy (such as light, mechanical force or magnetism), exogenous stimuli-responsive nanomaterials can undergo energy conversion or structural changes, thereby regulating cellular functions and accelerating wound healing. For example, light-responsive nanomaterials can generate heat energy through the photothermal effect 41, electro-responsive nanomaterials can generate electrical signals through the piezoelectric effect or the triboelectric effect 42-45, and magnetic-responsive nanomaterials can undergo macroscopic deformation or microscopic topological structure changes under the stimulation of a magnetic field, thereby generating mechanical signals for skin tissue cells 46. By adjusting the external energy stimulation parameters, exogenous stimuli-responsive nanomaterials can prevent harm to surrounding tissues and precisely control their therapeutic effects, thereby ensuring the safety and controllability of wound treatment 47.

Additionally, dual stimuli-responsive nanomaterials can respond to two distinct stimulus sources. These mainly include dual endogenous stimuli-responsive nanomaterials, dual exogenous stimuli-responsive nanomaterials, and endogenous-exogenous stimuli-responsive nanomaterials. By integrating two distinct response mechanisms, dual stimuli-responsive nanomaterials can adapt to more complex wound microenvironments and promote wound healing in multiple healing stages 48.

This review starts by exploring the types of stimulus sources and systematically reviews the mechanism, design strategies, and modification methods of various stimuli-responsive nanomaterials in promoting wound healing. Unlike previous reviews, this review focuses on analyzing how stimuli-responsive nanomaterials, by changing their own physical and chemical properties or by utilizing their own characteristics, exert multiple mechanisms, such as bactericidal, anti-inflammatory, immune regulatory, and promote tissue regeneration effects to accelerate wound healing. In addition, this review proposes corresponding solutions to the biological safety risks and scale-up problems faced by stimuli-responsive nanomaterials during application. The aim of this review is to provide scientific guidance and theoretical support for the design and clinical translation of stimuli-responsive wound treatment nanomaterials in the future.

## 2. Application of stimuli-responsive nanomaterials in wound healing

Stimuli-responsive nanomaterials can be mainly classified into endogenous stimuli-responsive nanomaterials, exogenous stimuli-responsive nanomaterials, and dual stimuli-responsive nanomaterials. It can exert multiple effects in accelerating the healing of bacterial-infected wounds, diabetic wounds, burn wounds and so on. Table 1 summarizes the material composition, mechanism of action, and application types of wounds of stimuli-responsive nanomaterials (Table 1).

## 3. Endogenous stimuli-responsive nanomaterials

Endogenous stimuli-responsive nanomaterials include pH-, enzyme-, ROS-, glucose-, ATP-, and GSH-responsive nanomaterials. These nanomaterials accelerate wound healing mainly through targeted delivery of therapeutic substances and by mimicking the activity of natural enzymes.

### 3.1. pH-responsive nanomaterials

**Figure 2:**
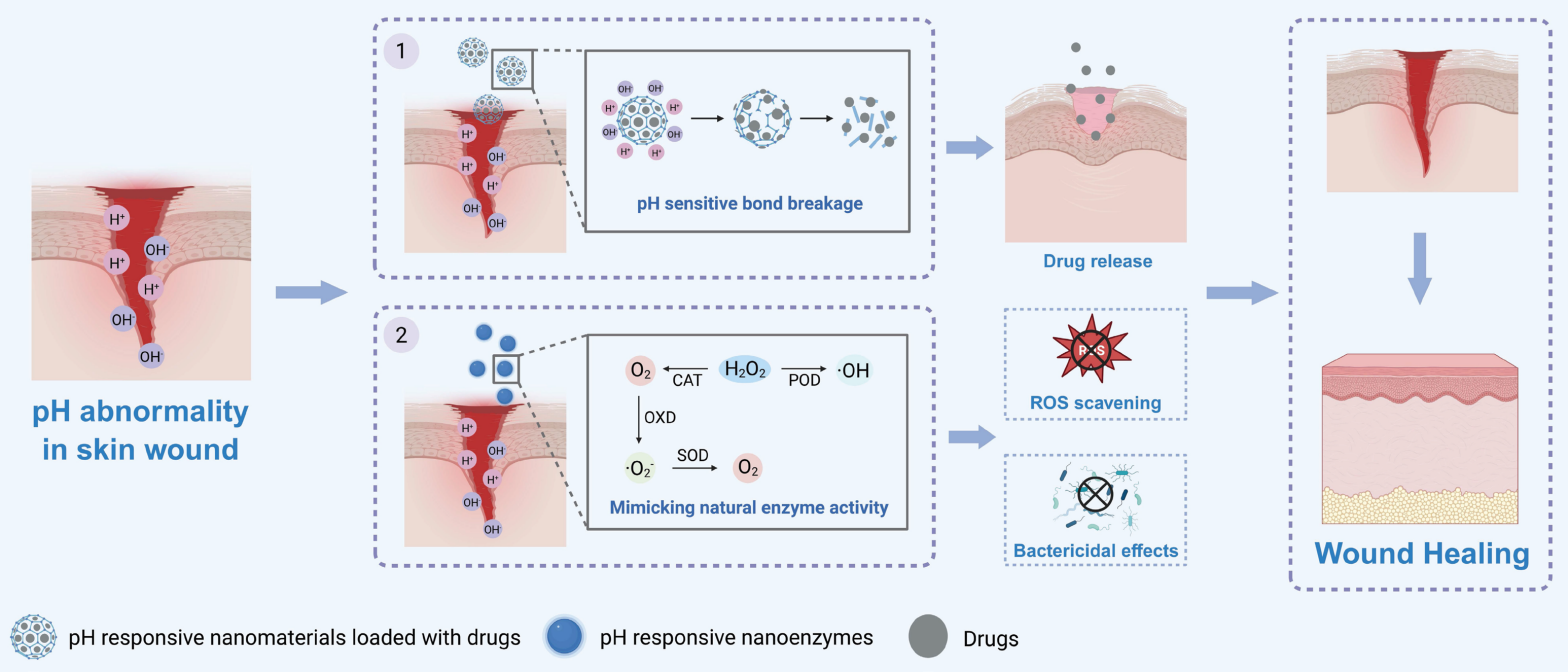
Diagrammatic illustration of the pH-responsive nanomaterials applied in wound treatment. The figure illustrates how these nanomaterials respond to pH changes in the wound microenvironment. Upon exposure to abnormal pH, the nanomaterials undergo structural changes to release therapeutic cargoes or mimic natural enzyme activity, thereby promoting wound healing. Created in https://BioRender.com.

The pH value undergoes significant changes during wound healing. Specifically, during the inflammatory stage, the pH is acidic, whereas during the proliferation stage and remodeling stage, it is weakly acidic to neutral 73. Therefore, researchers have designed acidic pH-responsive and alkaline pH-responsive nanomaterials to promote wound healing **(Figure 2)**.

When the wound pH is acidic, nanomaterials that respond to acidic pH can act through the following two mechanisms. The first mechanism involves structural disintegration to deliver therapeutic components. The second mechanism involves mimicking natural enzyme activities and thereby accelerating wound healing.

In acidic microenvironments, acidic pH-responsive nanomaterials can undergo structural disintegration and deliver therapeutic components, thereby accelerating wound healing 26, 49, 74-77. For instance, researchers developed an acidic pH-responsive nanosystem by modifying the surface of silver nanoparticles (Ag NPs) with ZIF-8 and then encasing allicin within the ZIF-8 framework. In the acidic wound microenvironment, H^+^ will react with the ZIF-8 framework through protonation interactions and cause structural disintegration of the nanosystem, thereby releasing Ag^+^ and allicin to exert antibacterial and antioxidant effects. In addition, ZIF-8 on the surface of Ag NPs can prevent their aggregation to lower toxicity, thereby accelerating the healing of bacterially infected wounds efficiently and safely 49, 78. Additionally, some researchers have developed acidic pH-responsive polyserotonin (PST)/Ag nanoparticles. In the acidic microenvironment of bacterially infected wounds, the PST shell layer of these nanoparticles breaks down and enables the release of Ag^+^. Then by inducing oxidative stress in bacteria, disrupting the bacterial membrane and affecting their metabolic pathways to exert bactericidal effects and accelerate skin wound healing 76, 79.

Furthermore, designing a dual acidic pH-responsive nanosystem can enhance the efficacy and safety of pH-responsive nanomaterials 80, 81. For instance, researchers have designed P-ZIF nanoparticles by loading polyhexamethylene biguanide (PHMB) into a ZIF-8 framework. Then, the dual pH-responsive HASPZ was developed by incorporating these nanoparticles via boronic ester bonds into a sodium alginate-based hydrogel. In the mildly acidic microenvironment of infected wounds, the boronic ester bonds break down 82, 83, releasing P-ZIF nanoparticles to exert a preliminary antibacterial effect. In a severely infected wound with a more acidic microenvironment, the P-ZIF nanoparticles further disintegrate to release PHMB, providing a potent antibacterial effect. Additionally, during the disintegration of P-ZIF nanoparticles, Zn^2+^ can be released to accelerate angiogenesis. This release pattern can prevent drug overdose to avoid side effects. Eventually, this nanohydrogel efficiently and safely promotes deep second-degree burn wound healing 80. Additionally, researchers have constructed a dual acidic pH-responsive nanomaterial system by loading α-phase manganese sulfide nanoparticles (α-MnS NPs) into a hydrogel crosslinked by Schiff base bonds. When the wound microenvironment is acidic, it triggers the hydrolysis of Schiff base bonds, leading to the release of α-MnS NPs. These α-MnS NPs subsequently degrade in the acidic microenvironment and release H_2_S, thereby exerting anti-inflammatory effects by reducing the expression of proinflammatory factors (such as IL-6 and IL-1β) to accelerate wound healing 81.

In addition to delivering therapeutic components, acidic pH-responsive nanomaterials can also mimic natural enzyme activity to accelerate wound healing 84-87. For example, researchers have used sulfated fucoidan as both a reducing agent and carrier to synthesize pH-responsive gold nanoparticles (Fuc@AuNPs). In the acidic wound microenvironment, a high concentration of H^+^ alters the surface charge distribution of AuNPs, thereby exhibiting oxidase (OXD)-like activity by promoting the adsorption of O_2_ on its surface and catalyzing the transformation of O_2_ into singlet oxygen (^1^O_2_), thus exerting a bactericidal effect. As the wound infection is controlled, the local pH gradually returns to the normal physiological level. At this stage, the OXD-like activity of Fuc@AuNPs is inhibited, and instead, the Fuc@AuNPs exhibit superoxide dismutase (SOD)-like activity to scavenge ROS while synergizing with the intrinsic anti-inflammatory properties of fucoidan to mitigate inflammatory responses and accelerate the healing of bacteria-infected wounds 87.

When the wound pH is alkaline, nanomaterials that respond to alkaline pH can act through the following two mechanisms. The first mechanism involves structural disintegration through covalent bond cleavage to deliver therapeutic components. The second mechanism involves mimicking natural enzyme activities and thereby accelerating wound healing.

On the one hand, in the alkaline wound microenvironment, the silicon‒oxygen bonds (Si‒O‒Si) in the silica nanoparticle (SiNP) framework can undergo nucleophilic substitution reactions with OH^-^ and break down. This process leads to the disintegration of SiNPs due to substrate etching and increased porosity. Afterward, the encapsulated chlorhexidine (CHX) is released to exert a bactericidal effect, thereby effectively treating chronic infected wounds 50.

On the other hand, alkaline pH-responsive nanomaterials can also act by mimicking natural enzyme activities to accelerate wound healing 88, 89. For instance, researchers developed a nanocomposite with multienzyme-like activity (Mo, Fe/Cu, I-Ag@GOx) and anchored it within a hydrogel. This nanocomposite operates through a “pH-switched glucose-initiated cascade reaction” to accelerate diabetic wound healing. In the hyperglycemic microenvironment of diabetic wounds, the nanocomposite first initiates the first cascade reaction relies on glucose oxidase (GOx), which catalyzes the decomposition of glucose and O_2_ into gluconic acid and H_2_O_2_ to consume excess glucose. The nanocomposite subsequently mimics peroxidase (POD)-like and OXD-like activities, converting H_2_O_2_ into superoxide anions (·O_2_^-^) and hydroxyl radicals (·OH), thereby exerting bactericidal effects. As inflammation diminishes and the local pH gradually increases to a mildly alkaline state, the activity of the nanocomposite switches to a second cascade reaction and simulates SOD-like activity to scavenge excess ROS and generate O_2_, thereby mitigating oxidative stress and tissue hypoxia 89.

### 3.2. Enzyme-responsive nanomaterials

**Figure 3:**
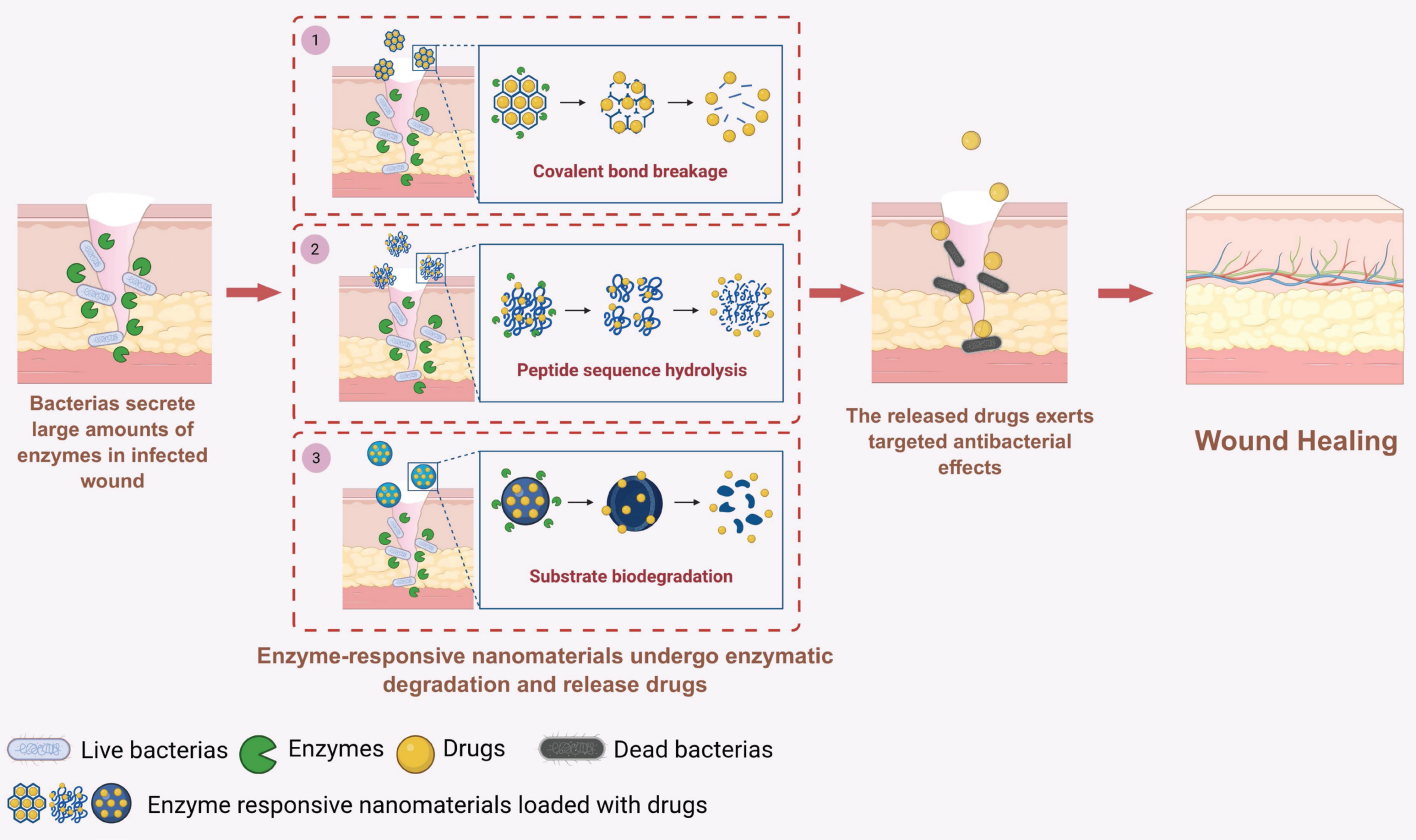
Diagrammatic illustration of the enzyme-responsive nanomaterials applied in wound treatment. The figure shows how these nanomaterials are designed to respond to specific enzymes that are overexpressed in the wound microenvironment. Upon encountering elevated levels of enzymes, the nanomaterials undergo triggered degradation, leading to on-demand release of therapeutic cargoes precisely at the wound site. Created in https://BioRender.com.

In the wound microenvironment, the abnormal overexpression of various enzymes (such as MMPs) often prolongs inflammation, resulting in delayed wound healing 90. To overcome this problem, researchers have designed various enzyme-responsive nanomaterials to precisely deliver therapeutic components and accelerate wound healing 91-94. These nanomaterials respond primarily to changes in local enzyme expression levels through three mechanisms: first, by the cleavage of enzyme-responsive covalent bonds within the nanomaterials; second, by the hydrolysis of enzyme-responsive peptide sequences within the nanomaterials; and third, by the biodegradation of macromolecular substrate components within the nanomaterials that correspond to the enzymes **(Figure 3).**

**Figure 4:**
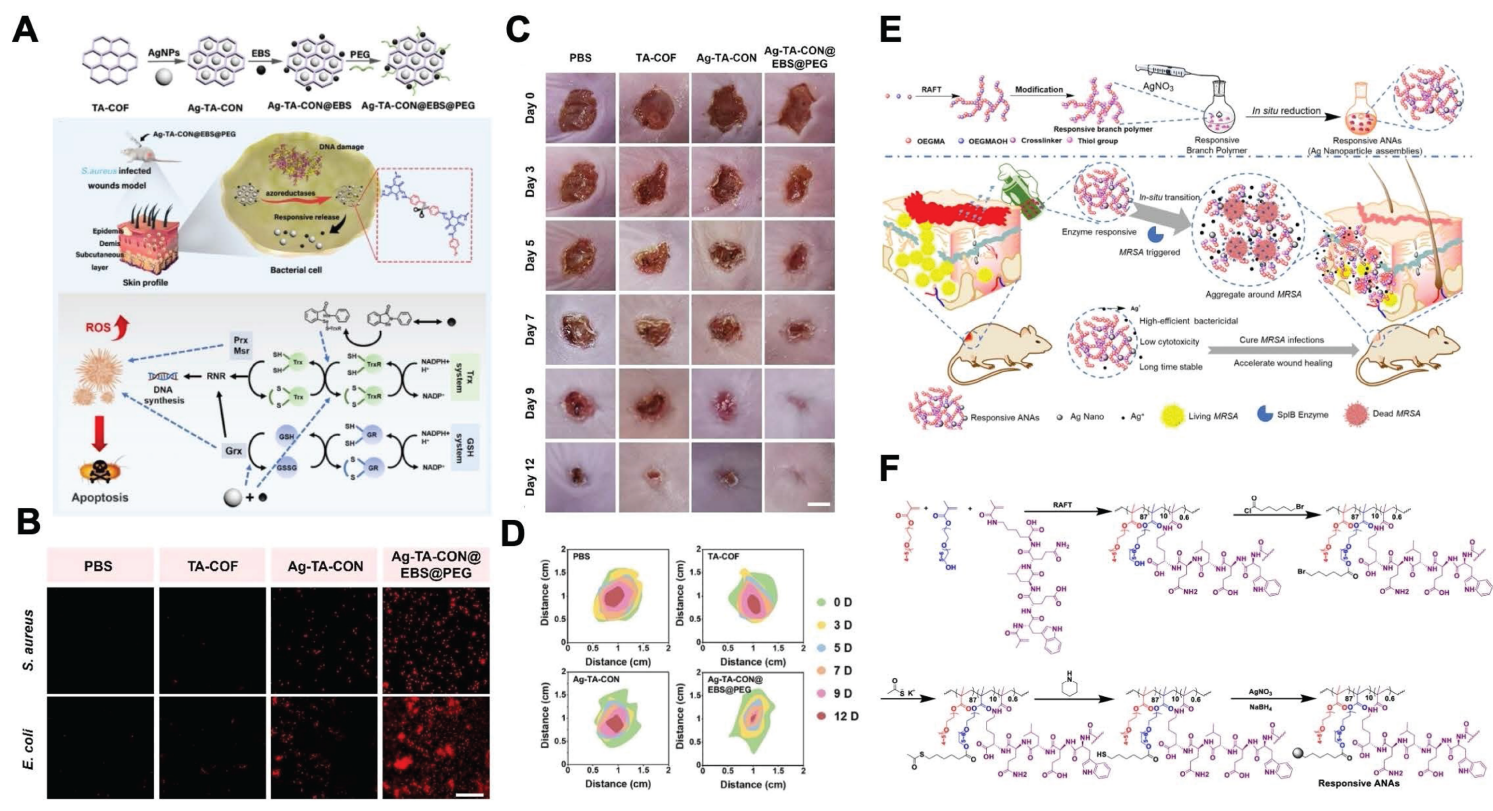
Enzyme-responsive nanomaterials for wound treatment. A) Diagrammatic illustration of the synthesis and antibacterial mechanism of Ag-TA-CON@EBS@PEG. B) Lethality fluorescence staining images of bacterial colonies on agar plates. C) Images of wound with different treatments at different times during the therapeutic process. D) Simulation analysis of skin wound healing process with different treatments. Adapted with permission from 33. Copyright 2022, American Chemical Society. E) Diagrammatic illustration of enzyme responsive ANAs for infected wound. F) Description of the synthesis of enzyme-responsive ANAs. Adapted with permission from 34. Copyright 2020, American Chemical Society.

First, enzyme-responsive nanomaterials can react with the high-enzyme wound microenvironment through covalent bond cleavage. Various bacteria involved in wound infections (such as *Escherichia coli* and *Staphylococcus aureus*) secrete azoreductase, leading to elevated expression levels of azoreductase in bacterially infected wounds. Therefore, covalent organic frameworks (COFs) containing azo bonds can cleave in response to high azoreductase levels in such wound microenvironments, enabling the targeted release of loaded ebselen (EBS) and silver ions to exert bactericidal and anti-inflammatory effects and thereby accelerating the healing of bacterially infected wounds **(Figure 4A-D)** 33.

Second, enzyme-responsive nanomaterials can achieve therapeutic component delivery through the hydrolysis of enzyme-sensitive peptide sequences. For example, the bacterium secretes large amounts of serine protease-like B enzyme proteins (SplB) in MRSA-infected wounds. On this basis, researchers have designed enzyme-responsive Ag nanoparticle assemblies incorporating the WELQK peptide sequence (methacrylate-tryptophan-glutamate-leucine-glutamine-lysine-methacrylate). The WELQK sequence can specifically respond to the SplB secreted by MRSA and then be hydrolyzed to release Ag nanoparticles. Eventually, this nanoassembly can exert bactericidal effects and promote the healing of MRSA-infected wounds **(Figure 4E–F)** 34. Additionally, in the diabetic wound microenvironment, the expression level of MMPs is elevated. Therefore, researchers have constructed MMP-responsive nanomaterials by incorporating MMP-cleavable peptide sequences into the nanofiber scaffold. In the high-MMP wound microenvironment, the MMP-responsive peptide sequences within the nanofiber scaffold undergo hydrolysis and deliver plasmids encoding human epidermal growth factor (hEGF), upregulate hEGF expression levels in fibroblasts and then accelerate the re-epithelialization process, thereby effectively promoting diabetic ulcer wound healing 51.

Finally, enzyme-responsive nanomaterials can also deliver therapeutic components through the enzymatic degradation of the corresponding macromolecular substrate 40, 95, 96. For instance, gelatin nanoparticles (Cur@Gel NPs), derived from gelatin, which is a substrate of MMPs, can undergo biodegradation in response to highly expressed MMPs in wounds, thereby releasing loaded curcumin to exert anti-inflammatory effects and accelerate wound healing 40. Moreover, hyaluronic acid (HA) can be electrostatically adsorbed onto the surface of a monolayer graphene quantum dot–carbon monoxide-releasing molecular nanocomplex (SGQDs-CORM), thereby constructing a hyaluronidase (HAase)-responsive nanomaterial, SGQDs-CORM@HA (SCH). At the site of bacterial infection, the HA on the surface of this enzyme-responsive nanomaterial can be specifically degraded by HAase secreted by MRSA, exposing the ultrathin nanosheet-structured SGQDs-CORM, which then exerts efficient bactericidal effects and accelerates the healing of bacterially infected wounds 95.

Currently, in the application of enzyme-responsive nanomaterials, the challenge of insufficient specificity of the enzyme-sensitive components still exists, which refers to the fact that the substrate recognition sequences of enzymes have some degree of overlap, such as those of proteases such as fibrinolytic enzymes and MMP. This overlap can easily lead to incorrect responses of enzyme-sensitive nanomaterials, causing cross-hydrolysis of enzyme substrates and subsequently resulting in the incorrect release of drugs in nontargeted wound microenvironments, significantly reducing therapeutic efficiency. To address this issue, future approaches can consider the use of phage display technology to design novel short peptide sequences that can specifically bind to the MMP. This peptide sequence can serve as a novel enzyme-sensitive element to effectively prevent erroneous release triggered by nontargeting enzymes.

In recent years, studies have quantitatively analyzed the targeting performance of “ultrahigh specificity” peptides obtained through “phage display technology” and *in vivo* validation has been completed in wound models. Common methods for quantifying affinity include isothermal titration calorimetry, surface plasmon resonance, and biolayer interferometry 97-100. For example, Liu *et al*. first screened a short peptide, KG7, that specifically targeted the bacterial biofilm protein PSMα1 from a twelve-peptide phage display library. Using the ITC method, they measured the dissociation constant of KG7 and PSMα1 and confirmed that its high affinity was approximately 66 times greater than that of the control peptide. The results showed that KG7 could bind to the key functional region of PSMα1 and interfere with the assembly of the biofilm matrix. Furthermore, researchers modified KG7 onto the surface of photothermal nanomaterials to construct a targeted nanoplatform. It was confirmed that this nanocomposite could accumulate at the biofilm site and exert a photothermal effect to destroy the biofilm, thereby accelerating diabetic infected wound healing 100. These studies have provided examples for the quantitative determination of the affinity of highly specific targeting peptides and *in vivo* functional validation.

### 3.3. ROS-responsive nanomaterials

**Figure 5:**
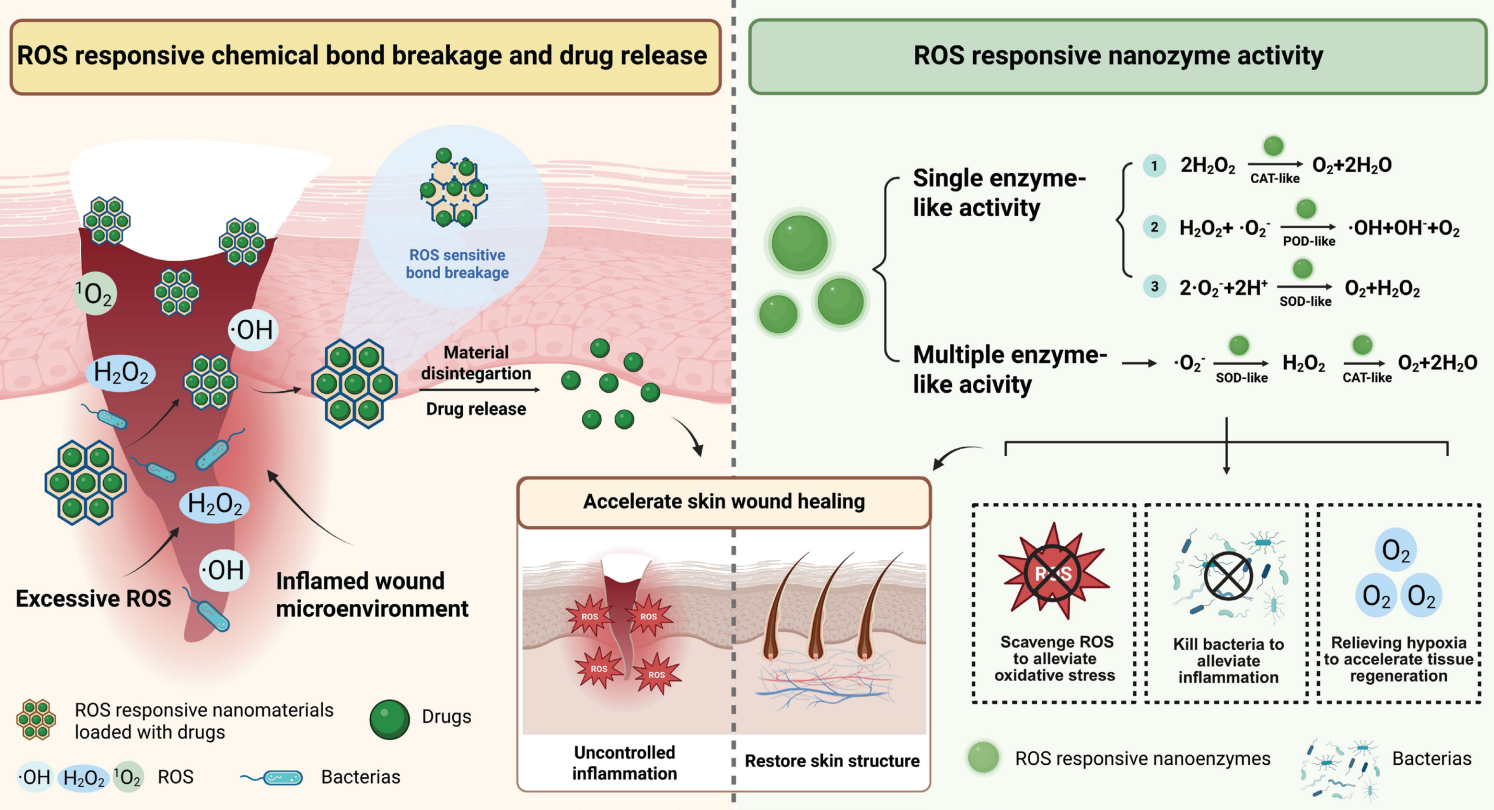
Diagrammatic illustration of the ROS-responsive nanomaterials applied in wound treatment. The figure illustrates how these nanomaterials respond to ROS changes in the wound microenvironment. Upon exposure to elevated ROS levels, the nanomaterials undergo structural changes to release therapeutic cargoes or mimic natural enzyme activity, thereby promoting wound healing. Created in https://BioRender.com.

Excessive ROS (such as H_2_O_2_, ·OH, and ·O_2_^-^) in the wound microenvironment can prevent the physiological transition from the inflammation stage to the proliferation stage, thereby delaying wound healing 101. ROS-responsive nanomaterials are nanomaterials that can detect and react to excessive ROS. They primarily accelerate wound healing through the following two mechanisms: (1) Chemical bonds within the nanomaterial react with ROS and cleavage, thereby triggering the release of loaded drugs. (2) ROS-responsive nanozymes that can detect and respond to ROS, mimicking single-enzyme or multiple-enzyme activities, thereby exerting bactericidal or anti-inflammatory effects **(Figure 5)**.

Firstly, ROS-responsive nanomaterials can deliver drugs through the cleavage of trisulfide bonds. For example, in the high oxidative stress microenvironment of diabetic wounds, lipid nanoparticles containing trisulfide bonds can respond to high levels of ROS, leading to the cleavage of trisulfide bonds and releasing encapsulated IL-4 mRNA, inducing macrophages polarizing toward anti-inflammatory phenotype and inhibiting excessive inflammation, thereby promoting diabetic wound healing 52. Furthermore, ROS-responsive nanomaterials can also trigger drug release by breaking the borate ester bonds. For example, in the high level of ROS microenvironment of diabetic wounds, nanoliposomes containing borate ester bonds will undergo oxidative cleavage, triggering the sustained release of loaded antimicrobial peptides and puerarin. These nanoliposomes can promote angiogenesis, exert antibacterial, anti-inflammatory effects, thereby accelerating diabetic wound healing 32.

Secondly, ROS-responsive nanozymes can mimic single-enzyme or multiple-enzyme activities, thereby exerting bactericidal, antioxidant effects and relieving tissue hypoxia to accelerate wound healing. On the one hand, ROS-responsive nanozymes that mimic single enzyme activities (such as CAT-like, POD-like, and SOD-like) can accelerate wound healing by exerting bactericidal or antioxidant effects 102-105. For example, researchers embedded lanthanide elements and transition metal ions into the lattice of iron-based nanozymes, reducing the free energy barrier of the catalytic reaction and facilitating electron transfer, thereby successfully constructing a high-entropy nanocrystalline structure nanozyme (C-ZnFeO@Nd NPs) with advanced POD-like catalytic ability. This nanozyme can respond to the high level of H_2_O_2_ at the infected wound site and catalyze H_2_O_2_ decomposition into ·OH. Subsequently, by disrupting the bacterial cell membrane and DNA, this nanozyme exerts bactericidal effect and accelerates bacterial-infected wound healing 103. In addition, ROS-responsive nanozymes that can mimic CAT-like or SOD-like activities can exert antioxidant effects by decomposing high concentrations of ROS in the wound microenvironment. For example, porphyrin-based bimetallic MOF nanoparticles with Mn-N_4_ active sites can catalyze the decomposition of excessive H_2_O_2_ in diabetic wounds and generate oxygen through their CAT-like activity. These nanoparticles can mitigate oxidative stress and tissue hypoxia, thereby accelerating wound healing 105. Furthermore, the Cu_2_Se nanosheets with SOD-like activity have been proven to be able to catalytically decompose O_2_·^-^ in the wound microenvironment, thereby relieving oxidative stress and promoting acute wound healing 104.

On the other hand, ROS-responsive nanoenzymes with the ability to simulate multi-enzyme activities can exert antioxidant and hypoxia-relieving effects to accelerate wound healing 105,106. For instance, cerium oxide-zoledronic acid nanozyme with surface-modified malic acid can accelerate diabetic wound healing by cascading the simulation of SOD-CAT-like activities. Firstly, the nanozyme responds to ROS through the redox cycle of Ce^3+^/Ce^4+^, exerting SOD-like activity and catalyzing the decomposition of ·O_2_^-^ at the wound site to generate H_2_O_2_; meanwhile, the Ce^3+^-related oxygen vacancies can enhance the efficiency of ROS adsorption and electron transfer, further improving its SOD-like activity. Additionally, by the stable binding and activation of H_2_O_2_, Ce^4+^ within the nanozymes can provide CAT-like activity, which catalyzes the decomposition of H_2_O_2_ to generate oxygen. By mitigating oxidative stress and tissue hypoxia in diabetic wounds, this strategy decomposes the potentially harmful intermediate H_2_O_2_, promotes macrophage polarization to the M2 phenotype and enhances angiogenesis, thereby promoting diabetic wound healing 106.

### 3.4. Glucose-responsive nanomaterials

**Figure 6:**
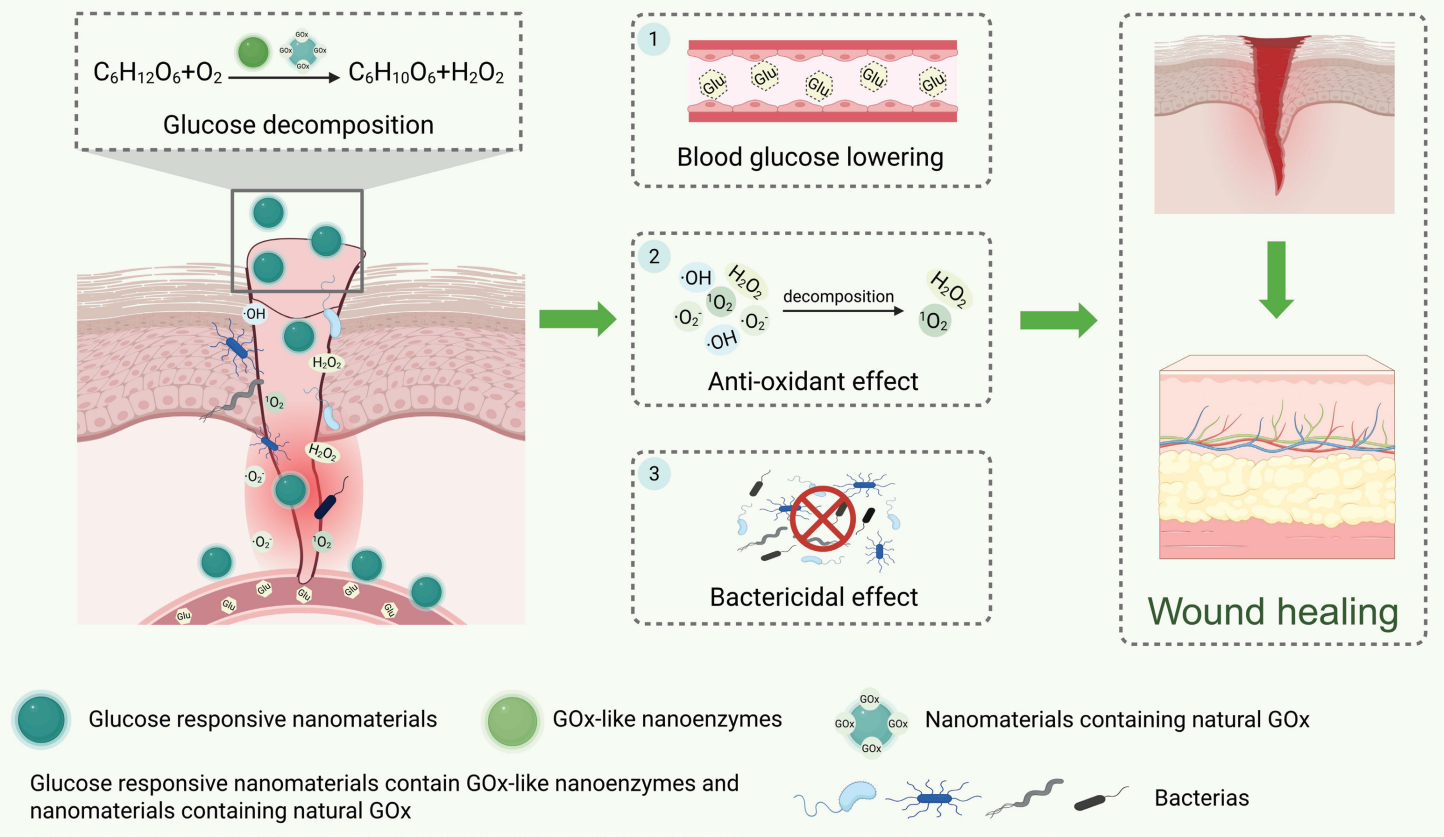
Diagrammatic illustration of the glucose-responsive nanomaterials applied in wound treatment. The figure shows how these nanomaterials are designed to respond to high glucose levels in diabetic wound microenvironments. Upon encountering elevated glucose, the nanomaterials can mimic GOx activity and exert certain effects, thereby promoting diabetic wound healing. Created in https://BioRender.com.

Hyperglycemia in diabetic wounds leads to a wound microenvironment characterized by persistent inflammation, impaired angiogenesis, and susceptibility to infection, thereby delaying wound healing 36, 107. GOx can convert glucose into gluconic acid and H_2_O_2_ to reduce glucose concentration 108. Thereby, designing glucose-responsive nanomaterials can target the hyperglycemic state of diabetic wounds to exert therapeutic effects 109,110. It is therefore the key component enabling glucose-responsive properties in nanomaterials. Currently, there are two primary strategies for designing glucose-responsive nanomaterials: the first is designing nanoenzymes that can mimic GOx activity. The second is incorporating natural GOx components into the nanomaterials. However, nanomaterials that can only mimic GOx activity will generate H_2_O_2_ and induce oxidative stress, thereby diminishing their therapeutic efficacy. Therefore, glucose-responsive nanomaterials exhibiting multi-enzyme-like activity or combined with other therapeutic agents are required for treating diabetic wounds **(Figure 6)**.

**Figure 7:**
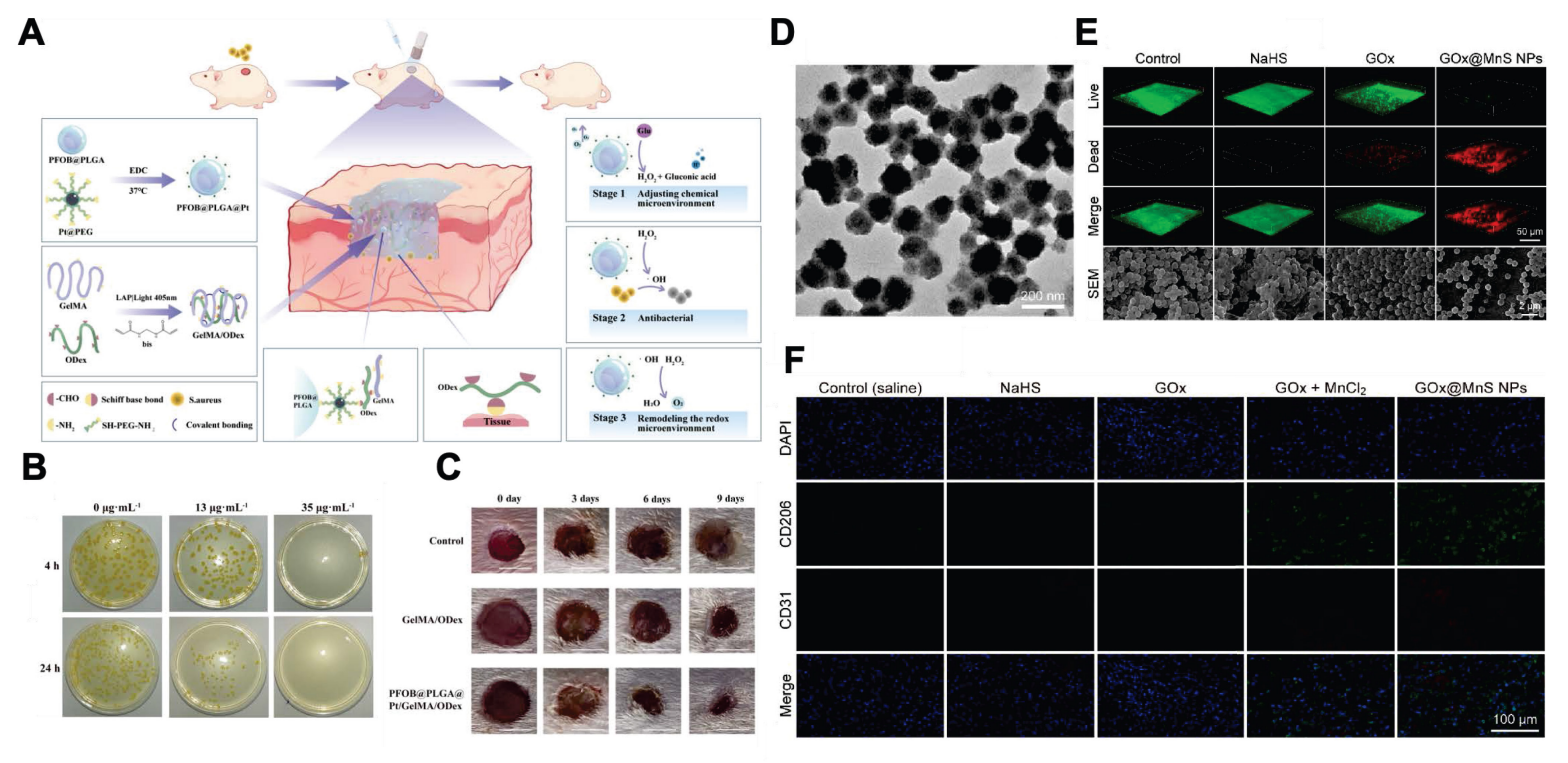
Glucose-responsive nanomaterials for wound healing. A) Diagrammatic illustration of PFOB@PLGA@Pt/GelMA/ODex nanohybrid hydrogel for diabetic infected wound. B) Images of bacteria colonies on agar plates incubated with nanohybrid hydrogels containing different Pt concentrations for different times. C) Images of wounds with different treatments at different times during the therapeutic process. Adapted with permission from 113. Copyright 2023, American Chemical Society. D) Transmission electron microscope (TEM) image of GOx@MnS nanoparticles. E) 3D CLSM and SEM images of GOx@MnS nanoparticles against MRSA. F) Angiogenesis and degree of inflammation in diabetic infected wound model with different treatments. Adapted with permission from 115. Copyright 2023, American Chemical Society.

Firstly, nanomaterials exhibiting multi-enzyme-like activity can trigger a cascade reaction through mimicking GOx-like activity first, thereby reversing multiple harmful factors in the diabetic wound microenvironment 111-113. For example, it has been reported that the platinum nanoparticles (Pt NPs) in the nanocomposite PFOB@PLGA@Pt/GelMA/ODex can mimic multi-enzyme activities, including GOx, OXD, and SOD. The Pt NPs first imitate GOx-like activity to lower the concentration of glucose. Then, it can mimic OXD and SOD activities to exert bactericidal and antioxidant effects, respectively, thus promoting diabetic wound healing **(Figure 7A-C)** 113.

Secondly, nanomaterials containing natural GOx components combined with gas therapy or drug delivery can also promote diabetic wound healing 110, 114, 115. For example, the GOx component of GOx@MnS nanoparticles can detect high glucose levels at diabetic wound sites and break them down to generate H_2_O_2_. It then initiates the reaction between MnS and H_2_O_2_, which generates H_2_S and ·OH to exert anti-inflammatory effects and bactericidal activity, thereby accelerating diabetic infected wound healing **(Figure 7D-F)** 115.

Based on the characteristics of natural enzymes, nanomaterials containing natural GOx have higher catalytic specificity and enzyme activity. However, their activity is easily influenced by factors such as pH and temperature in the wound microenvironment 116, 117. While the catalytic activity of the nanoenzyme mimicking GOx is weaker than that of natural GOx, it is less susceptible to other factors. Consequently, considering the features of these two kinds of glucose-responsive nanomaterials, we suggest that future material selection should consider blood glucose levels and the duration of treatment. For treating wounds with excessively high blood glucose levels that require rapid glucose reduction, the use of nanomaterials containing natural GOx components may be more appropriate. In contrast, for wounds with mildly raised blood glucose levels requiring sustained normal glucose levels, the durability of materials should be prioritized. Thus, nanoenzymes that mimic GOx activities may be the optimal selection.

Alongside GOx, introducing specific glucose-sensitive groups is another strategy to construct glucose-responsive nanomaterials. For example, introducing phenylboronic acid groups into nanoparticles and loading specific molecules or drugs within them can construct novel glucose-responsive nanomaterials. This kind of nanomaterial can sense the high concentration of glucose in diabetic wounds, and then initiate the react between the phenylboronic acid group and glucose, thereby causing the material to disintegrate, then release the loaded drugs and accelerate diabetic wound healing.

### 3.5. Other endogenous stimuli-responsive nanomaterials

Besides the commonly used pH, enzyme, ROS, and glucose-responsive nanomaterials mentioned previously, other endogenous stimuli-responsive (such as GSH and ATP) nanomaterials can also promote wound healing and are gaining more attention. However, overall, glutathione (GSH) and adenosine triphosphate (ATP)-responsive nanomaterials still present significant research potential.

GSH-responsive nanomaterials can respond to GSH through redox reactions, thereby exerting bactericidal or immunomodulatory effects to accelerate wound healing 56, 118-122. When bacterial infections occur, widespread cell necrosis will release intracellular GSH and accumulate at the wound site. As a natural antioxidant, GSH can increase bacteria’s resistance to oxidative bactericidal agents (such as ROS and RNS), which reduces the effectiveness of these agents 123. To solve this problem, researchers have designed nanocomposites that can produce ROS and decompose GSH simultaneously. For example, CuCo_2_O_4_ nanoflowers can mimic OXD and POD activities, which enable them to react with surrounding H_2_O_2_ and O_2_ to generate ROS. At the same time, this nanomaterial also exhibits glutathione peroxidase (GPx)-like activity to deplete GSH in the wound microenvironment through redox reaction. These nanoflowers can weaken bacteria’s ROS resistance to exert potent bactericidal effects, thereby promoting bacteria-infected wound healing 56. Additionally, GSH-responsive nanomaterials can also promote wound healing through regulating immune response. For instance, the nitro-quantum dots (N-CDs) in the MACNL (melanin@AuNPs@CPPO@N-CDs@L-menthol) nanocomposite can undergo a redox reaction with GSH to release NO, thereby increasing macrophage polarization to the M2 phenotype and exert immunomodulatory effects. Eventually, this nanomaterial can accelerate diabetic infected wounds healing through anti-inflammatory and angiogenesis-promoting effects 120.

ATP-responsive nanomaterials can accelerate wound healing through targeted drug delivery 124-126. In bacterial-infected wounds, a notable feature is the continuous release of ATP by pathogens, which enables ATP-responsive nanomaterials to target areas of bacterial accumulation 124. For example, ZIF-8 (a nanomaterial composed of Zn^2+^ and dimethylimidazole linked by coordinate bonds) will degrade in high-level ATP environments. This occurs because ATP exhibits a higher affinity for Zn^2+^ than for 2-methylimidazole, enabling ATP to replace the 2-methylimidazole units within ZIF-8 and causing its framework to collapse 127. Researchers designed ZIF-8 nanocomposites that encapsulate indole-3-acetic acid (IAA) based on this principle. In bacterially infected wounds, the ZIF-8 nanocomposites will degrade to release IAA, which accelerates wound healing through generating ROS to exert bactericidal effect 125. However, the ATP-responsive nanomaterials designed using the above methods have the drawback of inaccurate response, as they may also respond to other stimulus sources (for instance, ZIF-8 also demonstrates pH-responsiveness). This can cause ZIF-8 to degrade before reaching the desired site, thereby restricting the therapeutic effect. To address this issue, future research should try to construct nanomaterials incorporating ATP-specific aptamers. Aptamers are single-stranded nucleic acids that have distinct secondary and tertiary structures, and they can selectively bind to target molecules 128,129. In the future, one possible approach is that we can utilize the principle of base complementary pairing to integrate ATP aptamers and their complementary DNA into a three-dimensional nanostructure and then load drugs onto it. In wound microenvironments with high ATP concentrations, ATP can establish a more stable three-dimensional conformational alignment with the aptamer due to spatial structural complementarity, hydrogen bonding, base stacking, and electrostatic interactions. This interaction will disrupt the original three-dimensional nanostructure formed by the ATP aptamer and its complementary DNA, ultimately facilitating drug release and wound treatment.

## 4. Exogenous stimuli-responsive nanomaterials

### 4.1. Light-responsive nanomaterials

**Figure 8:**
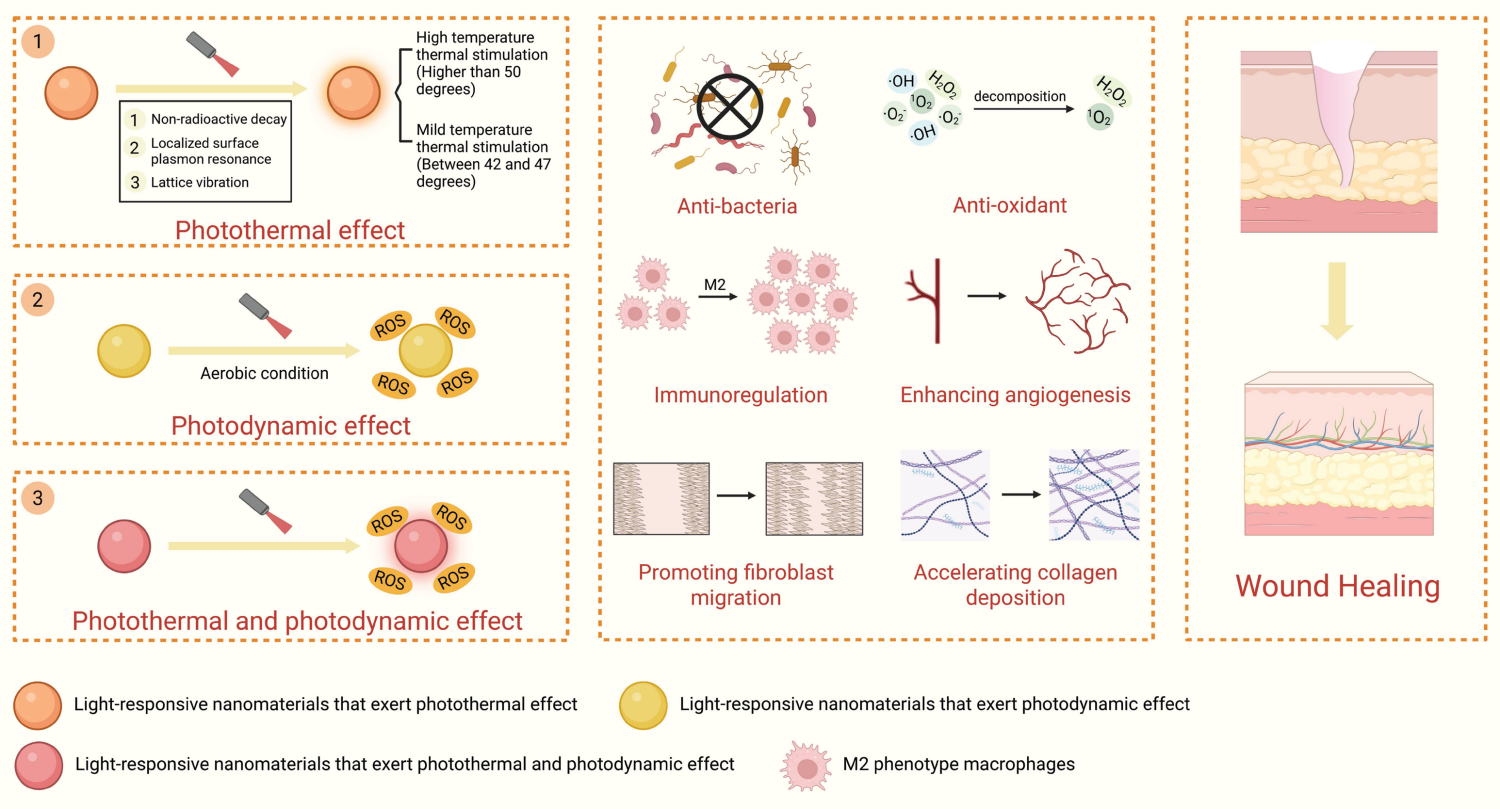
Diagrammatic illustration of the light-responsive nanomaterials applied in wound treatment. The figure illustrates the working principle by which these nanomaterials respond to external light stimuli. Upon light irradiation, the nanomaterials undergo photothermal conversion, photodynamic ROS generation, or exert photothermal-photodynamic synergistic effects to promote wound healing. Created in https://BioRender.com.

Light-responsive nanomaterials can detect and respond to external light stimuli and, in turn, exert photothermal effects, photodynamic effects, or photothermal-photodynamic synergistic effects (PTDT). This kind of nanomaterial can accelerate wound healing through bactericidal effect, regulating wound microenvironment, and promoting tissue regeneration **(Figure 8)**.

#### 4.1.1. Photothermal effect

Light-responsive nanomaterials can absorb specific wavelengths of light (such as visible and near-infrared light) and transform them into thermal energy through nonradiative decay 130. These nanomaterials can enhance wound healing through the following mechanisms: (1) exerting bactericidal effects; (2) modulating the wound microenvironment; and (3) promoting tissue regeneration.

**Figure 9:**
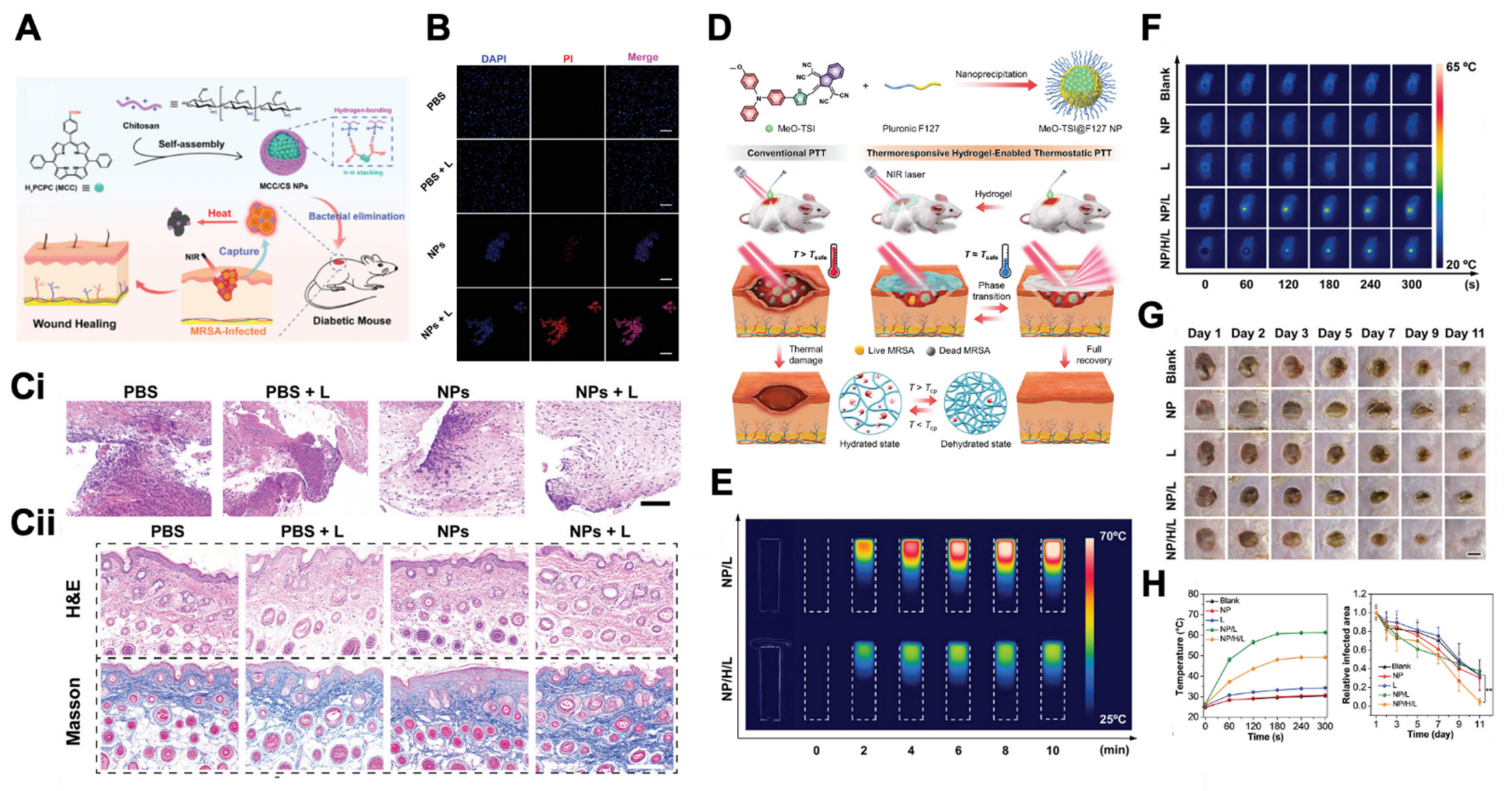
Light-responsive nanomaterials that exert photothermal effects for wound treatment. A) Diagrammatic illustration of MCC/CS nanoparticles for diabetic infected wound. B) Fluorescence images of MRSA with different treatments. C) H&E and Masson staining of the wound section with different treatment (Ci: Day 4, Cii: Day 14). Adapted with permission from 132. Copyright 2022, John Wiley and Sons. D) Diagrammatic illustration of the thermo-responsive hydrogel containing MeO-TSI@F127 nanoparticles for bacteria-infected wound. E) Thermal images of hydrogel containing MeO-TSI@F127 nanoparticles upon NIR irradiation. F) Thermal images of the bacteria-infected wounds with different treatment. G) Photographs of the bacteria-infected wounds with different treatment. H) Temperature profiles extracted from the corresponding thermal images and relative infected areas with different treatment. Adapted with permission from 146. Copyright 2023, John Wiley and Sons.

Light-responsive nanomaterials can exert bactericidal effects in two ways to promote wound healing. For one, the generation of high-temperature photothermal stimulation at temperatures higher than 50 °C directly causes thermal damage to bacterial structure 131,132. For instance, monocarboxylic phenol/chitosan nanoparticles (MCC/CS NPs) with positively charged surfaces can adsorb bacteria through electrostatic interactions. Under 606 nm near-infrared laser irradiation, the temperature of the MCC/CS NPs can subsequently reach 55 °C and further damage the phospholipid bilayer of the bacterial cell membrane, causing membrane rupture and the release of intracellular substances. Eventually, MCC/CS NPs can exert bactericidal effects and accelerate infected wound healing **(Figure 9A–C)** 132. As another example, molybdenum disulfide/chemically modified chitosan (MoS_2_@CSH) exhibits excellent photothermal properties and can exert POD-like activity. Under 808 nm near-infrared laser irradiation, the temperature of MoS_2_@CSH can increase to 51.9 °C, thereby exerting photothermal bactericidal effects. Additionally, it can simulate enzymatic activity similar to POD. Through a Fenton-like reaction, excessive H_2_O_2_ is broken down in the wound microenvironment and ·OH is generated, which has synergistic antibacterial effects and thereby accelerates infected wound healing by reshaping the wound microenvironment. In this example, the MoS_2_ nanosheets can stably combine with CSH because of their large specific surface area, thereby improving their dispersibility. This, in turn, enhances the photothermal conversion efficiency, enzyme-like activity, and biocompatibility 133.

For another, Light-responsive nanomaterials can also exert bactericidal effects by generating mild photothermal stimulation between 42 °C and 47 °C and combined with other mechanisms (such as antimicrobial ion release, chemodynamic therapy, etc.) to synergistically kill bacteria. This combined approach is necessary because mild thermal stimulation usually has only an inhibitory effect on bacteria rather than directly killing them. Therefore, it needs to be combined with other mechanisms to exert a bactericidal effect 134-136. For instance, a composite QPQH hydrogel dressing can be developed by loading polydopamine-coated zinc oxide nanoparticles (PDA@ZnO NPs) into a hydrogel containing quercetin (QT) and quaternary ammonium chitosan (QCS). Under 808 nm near-infrared laser irradiation, the PDA@ZnO NPs can generate mild thermal stimulation at approximately 45 °C, which can synergize with the slowly released Zn^2+^ to disrupt bacterial membrane integrity and metabolic balance. Simultaneously, the cationic groups of QCS exert electrostatic attraction and membrane-disruptive effects on bacteria, resulting in a combined bactericidal effect to accelerate infected wound healing 134. Additionally, light-responsive nanomaterials can also cooperate with chemodynamic therapy (CDT) to achieve bactericidal effects. For instance, researchers have fabricated Cu_X_O@PDA nanoparticles (CP NPs) capable of promoting wound healing through mild-temperature photothermal stimulation combined with POD-like activity. Under 808 nm near-infrared laser irradiation, CP NPs can generate mild thermal stimulation of approximately 45 °C that can disrupt bacterial metabolism. Moreover, mild thermal stimulation can also increase POD-like activity through reducing the energy required for the catalytic reaction, thereby accelerating ROS production to exert a chemodynamic bactericidal effect. Eventually, CP NPs can efficiently accelerate infected wound healing 135.

Second, light-responsive nanomaterials can accelerate wound healing through modulating wound inflammatory microenvironment 137,138. Under 808 nm near-infrared laser irradiation, the graphene oxide nanoparticles can generate mild thermal stimulation of 45 °C to increase the expression levels of the anti-inflammatory cytokines IL-4 and IL-10 in macrophages. This promotes their polarization toward the M2 phenotype to exert anti-inflammatory effects and accelerate wound healing 137.

Finally, light-responsive nanomaterials can accelerate wound healing by promoting tissue regeneration 139,140. For instance, under 808 nm laser irradiation, Prussian blue nanoparticles can generate a mild photothermal stimulus of 41 °C, upregulating hypoxia inducible factor-1α (HIF-1α) expression in human umbilical vein endothelial cells (HUVECs) and further promoting VEGF secretion, thereby enhancing cellular angiogenesis to accelerate diabetic wound healing 139. Additionally, under 908 nm laser irradiation, CuS@BSA nanoparticles can generate a mild thermal stimulus of 47 °C and upregulate the expression level of vimentin in mesenchymal stem cells (MSCs), thereby promoting MSC proliferation and increasing the fibroblast differentiation rate, accelerating collagen fiber deposition, and facilitating wound healing 140.

The photothermal temperature threshold includes the bactericidal temperature threshold and the tissue damage threshold. Specifically, the temperature generated by the photothermal effect can exert a bactericidal effect when it exceeds 50 °C, and the temperature threshold that can cause damage to healthy skin tissue is approximately 44 °C 141,142. High-temperature photothermal effects are often used to treat bacteria-infected wounds. These effects can rapidly sterilize by destroying bacterial structure through heat, but the heat generated can cause thermal damage to surrounding healthy tissues. Studies have shown that the damage to the skin caused by a temperature of 44 °C for 6 h is essentially equivalent to that caused by a temperature of 55 °C for 30 s 143. These findings suggest that in addition to temperature, the duration of action is another important factor for determining the degree of tissue damage. Therefore, when high-temperature photothermal effects are used to promote skin wound healing, the duration of action should be precisely controlled to prevent thermal damage to the surrounding tissues.

To address the aforementioned issue, in addition to controlling the duration of action, the “photothermal temperature wall” theory can also be utilized to precisely control the temperature 144. Specifically, when the temperature reaches a preset threshold, the color or transparency of the light-responsive nanomaterial can change reversibly, thereby reducing or blocking light absorption. This process will terminate the photothermal conversion and prevent excessive temperature increase. One approach is to combine light-responsive nanomaterials with thermochromic materials. For example, by using spiro lactone, myristic acid, bisphenol A (BPA), and a hollow-structured silica nanocarrier, researchers have constructed a temperature-controlled smart nanoparticle (TCSN). In the solid myristic acid without light irradiation, SL receives a proton from BPA to form SL-H^+^ and adopt an NIR-absorbing colored state. Under NIR light irradiation, when the temperature reaches the melting point of the myristic acid, SL-H^+^ loses a proton from BPA to adopt an NIR-transparent colorless state. Therefore, this nanoplatform can precisely control the temperature around the melting point (49 °C) of myristic acid and safely accelerate the healing of bacteria-infected wounds without damaging surrounding skin tissue 145. Another approach is to utilize the phase transition property of the hydrogel. When the temperature reaches the phase transition temperature of the hydrogel, it dehydrates and transforms into a white solid state, creating numerous light-scattering centers, thereby inhibiting further heating. For instance, the thermosensitive hydrogel P (NIPAM-AM) loaded with light-responsive MeO-TSI@F127 nanoparticles can be used to accelerate the healing of bacterially infected wounds without damaging surrounding skin tissue. Under 808 nm laser irradiation, MeO-TSI@F127 nanoparticles can generate thermal stimulation to kill bacteria. When the temperature reaches the phase transition temperature of P (NIPAM-AM), the PNIPAM undergoes a phase change to control the temperature, maintaining the photothermal equilibrium temperature between 45 and 50 °C **(Figure 9D-H)** 146.

High-temperature photothermal effects and mild-temperature photothermal effects can be switched by changing the incident light wavelength, frequency, and duration (Table 2). Therefore, in the future, on the basis of the different types of wounds and different healing stages, an optimal photothermal effect mode can be selected to treat wounds in a personalized manner. The following factors may be important to consider for the selection of different photothermal modes. From the perspective of wound types, the high-temperature mode, owing to its antibacterial properties, may be more suitable for infected wounds. Considering that diabetic wounds are characterized by both susceptibility to infection and impaired vascularization, the combined photothermal mode of high- and mild-temperature photothermal therapy may result in better therapeutic effects. During the wound healing stage, the high-temperature mode affects mainly the inflammatory stage through its antibacterial effect, whereas the mild-temperature mode can promote cell proliferation, differentiation and vascular regeneration, which is more suitable for the proliferative and remodeling stages. Therefore, the high-temperature mode is more suitable for the early stage of wound healing, whereas the mild-temperature mode is more appropriate for the later stage.

#### 4.1.2. Photodynamic effect

When activated by light in aerobic environments, light-responsive nanomaterials (nanophotosensitizers) can generate high levels of ROS through photodynamic effects 155, thereby exerting bactericidal effects and promoting wound healing.

**Figure 10:**
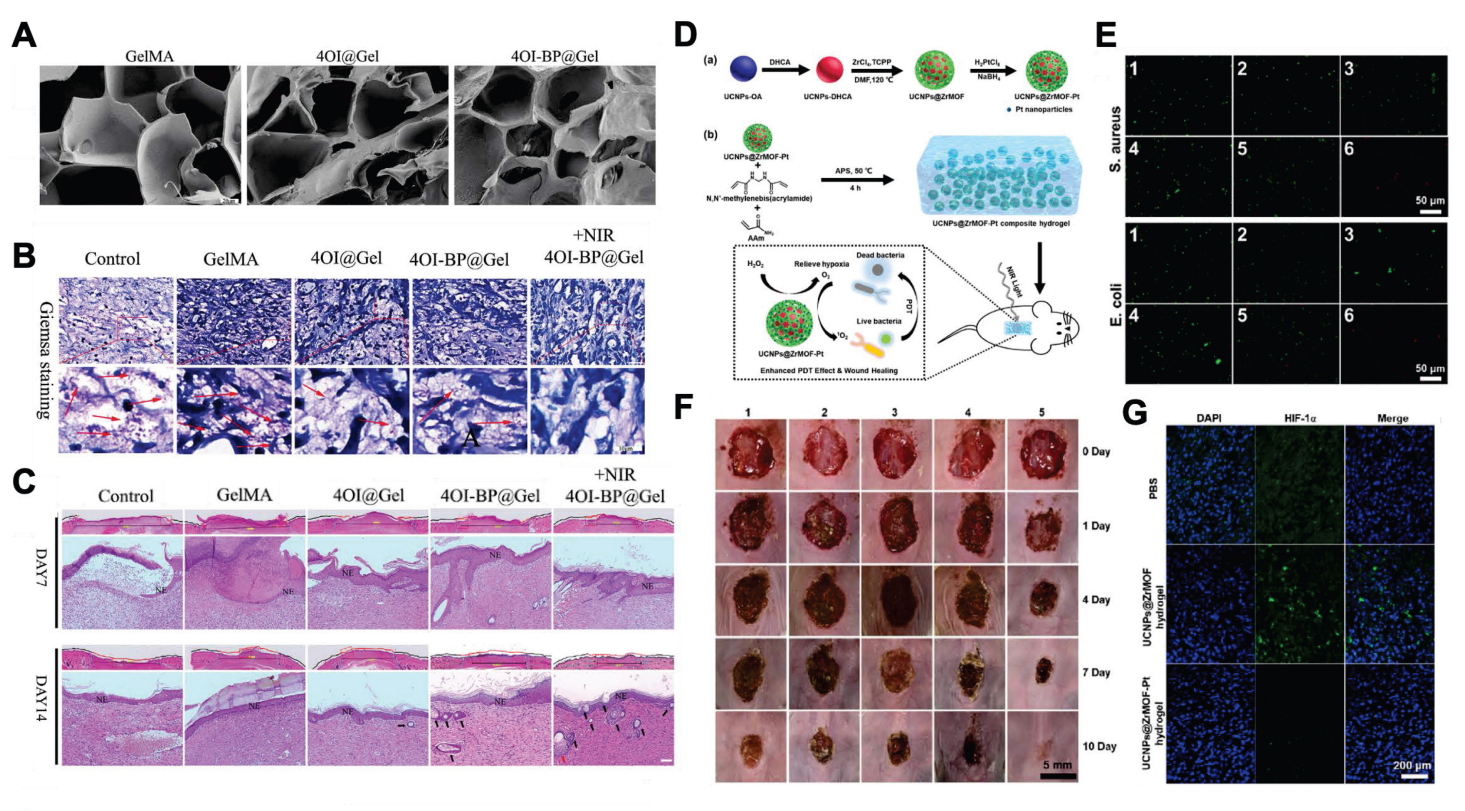
Light-responsive nanomaterials that exert photodynamic effects for wound treatment. A) SEM images of the GelMA, 4OI@Gel and 4OI-BP@Gel hydrogel. B) Giemsa staining of bacteria infected wound tissues after 1 week treatment. Red arrows show the bacteria. C) H&E staining of the wound section at the DAY7 and DAY14. Adapted with permission from 157. Copyright 2023, Elsevier. D) Description of the preparation of UCNPs@ZrMOF-Pt composite hydrogel and its antibacterial mechanism. E) Fluorescence images of live/dead bacteria after different treatment (1. PBS, 2. UCNPs@ZrMOF-Pt hydrogel, 3. UCNPs@ZrMOF + Laser, 4. UCNPs@ZrMOF-Pt + Laser, 5. UCNPs@ZrMOF + H_2_O_2_ + Laser, 6. UCNPs@ZrMOF-Pt + H_2_O_2_ + Laser). F) Fluorescence images of live/dead bacteria after different treatment (1. PBS, 2. Laser, 3. UCNPs@ZrMOF-Pt hydrogel, 4. UCNPs@ZrMOF hydrogel+ Laser, 5. UCNPs@ZrMOF-Pt hydrogel + Laser). G) Fluorescence images of bacteria-infected wound tissue with different treatment 169. Adapted with permission from Copyright 2022, John Wiley and Sons.

Nanophotosensitizers can undergo energy level transitions and transfer electrons and energy to nearby molecules, thereby generating high concentrations of ROS to exert bactericidal effects. Research has shown that MOF-based nanomaterials, black phosphorus nanosheets, and graphene quantum dots doped with halogens and nitrogen atoms can generate ROS through the photodynamic effect, thereby promoting wound healing through bactericidal and anti-inflammatory effects. For instance, researchers have embedded 4-octyl itaconate (4OI)-modified black phosphorus nanosheets into hydrogels. Under 808 nm laser irradiation, black phosphorus nanosheets can generate ROS to exert bactericidal effects. Moreover, the large specific surface area of black phosphorus nanosheets effectively increased the loading amount of 4OI. In the absence of laser irradiation, black phosphorus nanosheets can act as carriers to control the release of 4OI and exert anti-inflammatory effects, thereby accelerating the healing of diabetic ulcer wounds. In addition, as a degradable two-dimensional material, black phosphorus can gradually be oxidized and degraded into nontoxic phosphate ions under physiological conditions, avoiding long-term material residue. This significantly reduces the risks of chronic toxicity and foreign body reactions. In combination with its excellent light-responsive properties and antibacterial performance, it has unique application advantages in the field of wound healing **(Figure 10A-C)** 156-159.

The hypoxic microenvironment of chronic wounds and the fact that certain nanophotosensitizers can only be activated by visible light are two major factors that limit the production of ROS. To increase the efficiency of ROS generation by nanophotosensitizers and promote their bactericidal function 160-163, given the high H_2_O_2_ concentration in chronic wound microenvironments 164, researchers have developed nanophotosensitizers that simultaneously exert photodynamic effects and exhibit CAT-like activity, such as atomically dispersed Fe-doped oxygen-deficient molybdenum oxide MoO_3-X_ (ADFM) and Se@CeO_2_ nanoparticles. Such nanomaterials can decompose H_2_O_2_ to generate O_2_, thereby alleviating the hypoxic state and improving the efficacy of photodynamic reactions 165,166. Additionally, some nanophotosensitizers can be activated only by visible light, which has limited tissue penetration, thus limiting their application in deep wounds 167,168. To overcome this problem, researchers have combined nanophotosensitizers with upconversion nanoparticles (UCNPs) that can convert penetrating near-infrared light into visible light 169,170. For example, incorporating UCNPs with MOF-based nanophotosensitizers and modifying with platinum nanoparticles can simultaneously increase the photodynamic efficacy and address the problem of penetration depth. Under NIR irradiation, the UCNPs can transform the penetrating NIR laser into visible light and, in turn, activate the nanophotosensitizers, producing ^1^O_2_ to exert bactericidal effects through a photodynamic reaction. Moreover, the CAT-like activity of platinum nanoparticles can transform high levels of H_2_O_2_ into O_2_, thereby increasing the photodynamic antibacterial efficacy and accelerating wound healing **(Figure 10D-G)** 169.

However, most nanophotosensitizers cannot specifically target pathogens. Therefore, the high levels of ROS they generate spread throughout the entire illuminated area, damaging normal tissue cells at the wound edge and negatively impacting wound healing. To address this issue, a possible strategy can involve providing nanophotosensitizers with the ability to target pathogens. For example, nanophotosensitizers can be gathered around bacteria using biomimetic strategies, such as modifying the surface of nanomaterials with aptamers that specifically recognize bacteria (e.g., aptamer Apc for *Escherichia coli* 171 and aptamer SA31 for *Staphylococcus aureus* 172) or applying the bacterial cell membrane coating technique. This will enable the generated ROS to target bacteria more accurately, increasing both the bactericidal efficacy and the protection of surrounding tissue, thereby effectively and safely promoting wound healing.

#### 4.1.3. Photothermal-photodynamic synergistic effect

Nanomaterials that generate PTDT effects can accelerate wound healing through enhanced bactericidal activity and the activation of repair-related mechanisms. On the one hand, through photodynamic processes, light-responsive nanomaterials can facilitate the production of large amounts of ROS and oxidatively damage bacterial cell membranes. On the other hand, through photothermal processes, light-responsive nanomaterials can generate localized hyperthermia that directly increases bacterial cell membrane permeability and destroys bacterial biofilms. This synergistic effect makes it easier for ROS to pass through cell membranes and exert an oxidizing bactericidal effect. Additionally, PTDT effects lower the temperature and amount of ROS needed for bactericidal activity, thereby protecting skin tissue around the wound 170, 173-176. For instance, in bacteria-infected wounds, under 400 nm visible light irradiation, PAM-PDA/Ag@AgCl nanomaterials can simultaneously release thermal energy and generate ROS through the PTDT effect, thereby efficiently promoting the healing of infected wounds while avoiding damage to surrounding healthy tissue. Additionally, light-responsive nanomaterials can activate repair-related mechanisms to accelerate tissue regeneration. For example, under 808 nm laser irradiation, the CaSiO_3_-ClO_2_ @PDA-ICG nanoparticles (CCPI NPs) can exert a PTDT effect to activate AMPK signaling in macrophages and promote their polarization toward the M2 phenotype, thereby increasing transforming growth factor beta 1 (TGF-β1) secretion. Subsequently, it can activate epithelial‒mesenchymal transition (EMT) in tissue cells near the periphery of the wound, which significantly increases fibroblast migratory ability and accelerating granulation tissue formation and wound closure 177.

### 4.2. Electro-responsive nanomaterials

Electro-responsive nanomaterials refer to nanomaterials that can generate specific electrical responses to external mechanical stimuli (such as friction, physiological motion, ultrasound, magnetic force, etc.) and transform mechanical energy into electrical signals. Electro-responsive nanomaterials mainly include piezoelectric nanomaterials and triboelectric nanomaterials 178-181.

#### 4.2.1. Piezoelectric nanomaterials

**Figure 11:**
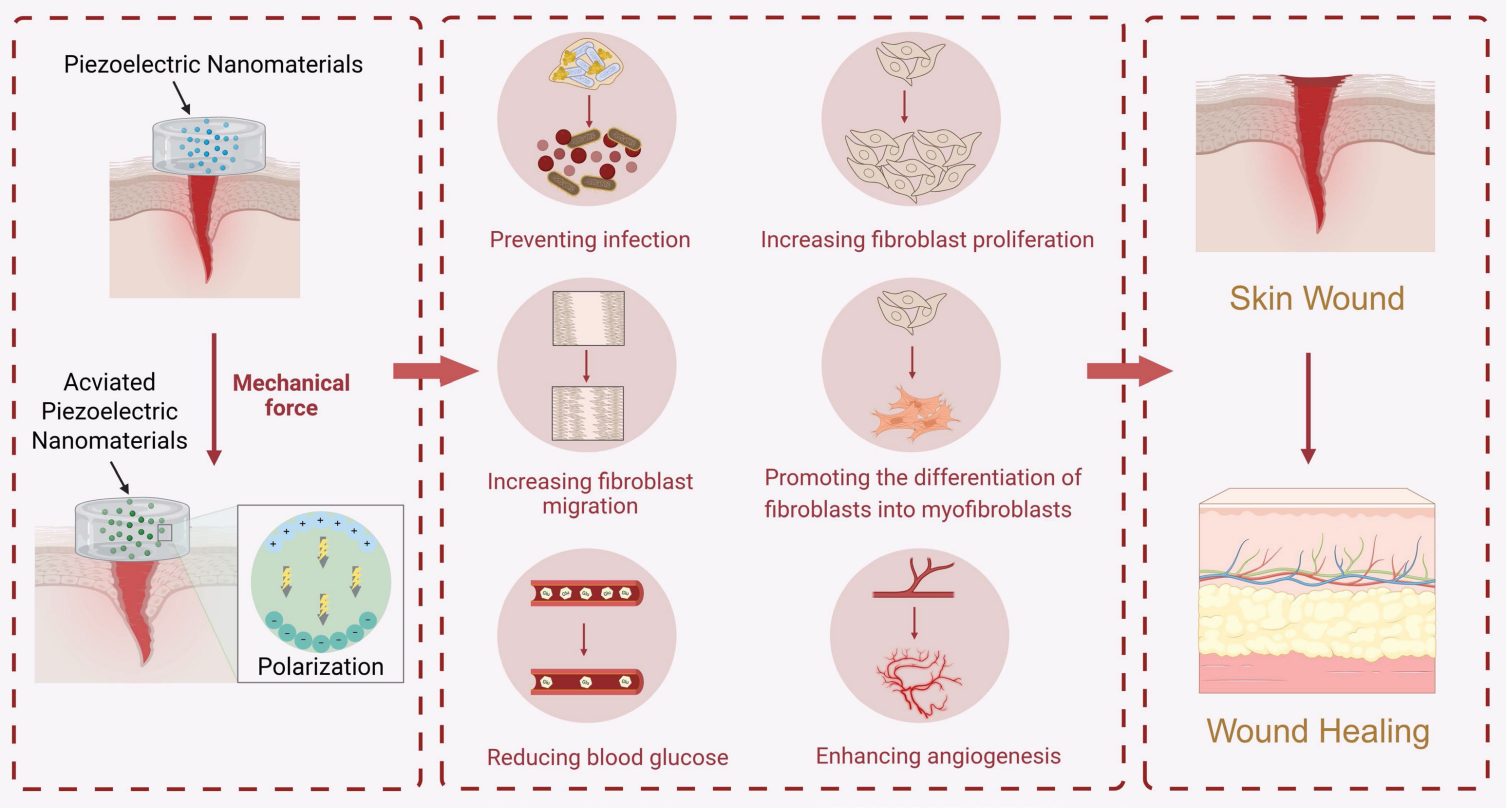
Diagrammatic illustration of the piezoelectric nanomaterials applied in wound treatment. The figure illustrates how these nanomaterials convert mechanical energy into electrical signals via the piezoelectric effect, thereby accelerating wound healing. Created in https://BioRender.com.

Piezoelectric nanomaterials have noncentrosymmetric crystal structures. Under external mechanical force stimulation, these materials will undergo internal polarization and generate electrical signals. They can accelerate wound healing by modulating the wound microenvironment; promoting cell proliferation, migration, and differentiation; and exerting bactericidal effects **(Figure 11)**.

Piezoelectric nanomaterials can mimic natural enzyme activity to modulate the wound microenvironment 11,182-184. For instance, in diabetic wounds, by integrating hyaluronic acid-encapsulated L-arginine, ultrasmall gold nanoparticles, and Cu_1.6_O nanoparticles loaded with phosphorus-doped graphitic carbon nitride nanosheets, researchers have constructed a piezoelectric ACPCAH nanocomposite, which can be activated by ultrasound and alkaline wound microenvironment. By regulating the surface electron transfer process, it can cascade mimics of the enzymatic activities of SOD-CAT-GOx-POD/nitric oxide synthase (NOS), thereby reducing blood sugar levels, exerting anti-inflammatory and bactericidal effects, alleviating hypoxia, and promoting vascular regeneration. Eventually, this piezoelectric nanocomposite can accelerate wound healing by modulating the diabetic wound microenvironment 183.

Furthermore, piezoelectric nanomaterials can generate electrical signals to promote cell proliferation, migration, and differentiation 63,185,186. For instance, when the exudate from a wound is absorbed, a ZnO nanoparticle-modified polyvinylidene fluoride/sodium alginate piezoelectric hydrogel (ZPFSA) can undergo vertical swelling. During physiological movement, it generates horizontal friction with the skin. Both of the aforementioned actions can provide mechanical stress to piezoelectric hydrogels, thereby generating electrical stimuli that promote fibroblast proliferation and migration to accelerate wound healing 63. Additionally, electrical stimulation generated by piezoelectric nanomaterials can regulate cell differentiation. For example, an electrically stimulated “Lock-ON/OFF” drug delivery system can be constructed by loading carboxylated carbon nanotubes/vancomycin hydrochloride (c-MWCNTs-VAN) onto a composite membrane composed of polyvinylidene fluoride (PVDF) and polyethylene oxide (PEO). This system can respond to mechanical stress generated by physiological movement and generate electrical stimulation through the piezoelectric effect. On the one hand, electrical stimulation can precisely control the on-demand release of VAN to exert its bactericidal effect. On the other hand, it can promote fibroblast proliferation and migration and myofibroblast differentiation, thereby accelerating wound closure 185. As another example, under the stimulation of a rotating magnetic field, the PCL/Ti_3_C_2_T_x_ MXene nanofiber membrane can generate an approximately 10.8 μA microcurrent. By activating and upregulating calcium signaling, it drives calcium influx, thereby regulating the migration, proliferation and differentiation of fibroblasts and accelerating the healing of diabetic wounds 187.

Finally, piezoelectric nanomaterials can also exert bactericidal effects by generating oxidative substances to accelerate wound healing 179,188,189. For instance, under ultrasound stimulation, piezoelectric C_3_N_4_ nanosheets can generate H_2_ and holes, thereby disrupting the electron transfer chain of bacteria and blocking their respiration to achieve bactericidal effects, thus promoting infected diabetic wound healing 189.

Improving the mechanical-electrical energy conversion efficiency of piezoelectric nanomaterials or their interactions with cells/bacteria is one possible way to enhance the efficacy of piezoelectric nanomaterials in treating skin wounds. This process involves three primary strategies: (1) modulating the internal structure of nanomaterials; (2) adjusting exogenous mechanical forces; and (3) combining other therapeutic approaches.

First, constructing a specific “Schottky junction” structure in piezoelectric nanomaterials can enhance their ability to produce oxidizing substances for bactericidal effects 190. For instance, the deposition of Au nanoparticles onto the surface of barium titanate (BT) nanocubes can form a “Schottky barrier” structure. By inhibiting electron‒hole recombination and promoting electrochemical reactions, compared with single-component piezoelectric nanomaterials, composite piezoelectric nanomaterials have demonstrated superior oxidizing substance generation and have exhibited enhanced efficacy in promoting infected wound healing 191.

Second, different mechanical stress parameters result in different levels of electrical stimulation from piezoelectric nanomaterials 192. Therefore, modifying mechanical stress conditions can regulate the effects of piezoelectric nanomaterials. For example, altering the intensity and duration of ultrasound exposure can produce distinct effects, such as antibacterial activity and promotion of tissue regeneration. These strategies can be applied during different phases of wound healing. For example, after 5 min of exposure to 1.5 W/cm^2^ ultrasound, barium titanate@macrophage membranes preactivated by *Staphylococcus aureus* (BTO@MMSa) can generate oxidative substances and exert bactericidal effects, thereby acting on the inflammatory phase of wound healing. While following 1 min of 0.8 W/cm^2^ ultrasound exposure, BTO@MMSa can act during the tissue regeneration phase of wound healing. It can upregulate the expression levels of repair-related genes such as VEGF, COL-I, and COL-III in fibroblasts, thereby promoting cell migration. The sequential application of high- and low-power ultrasound to infected wounds significantly accelerates the healing process 61.

**Figure 12:**
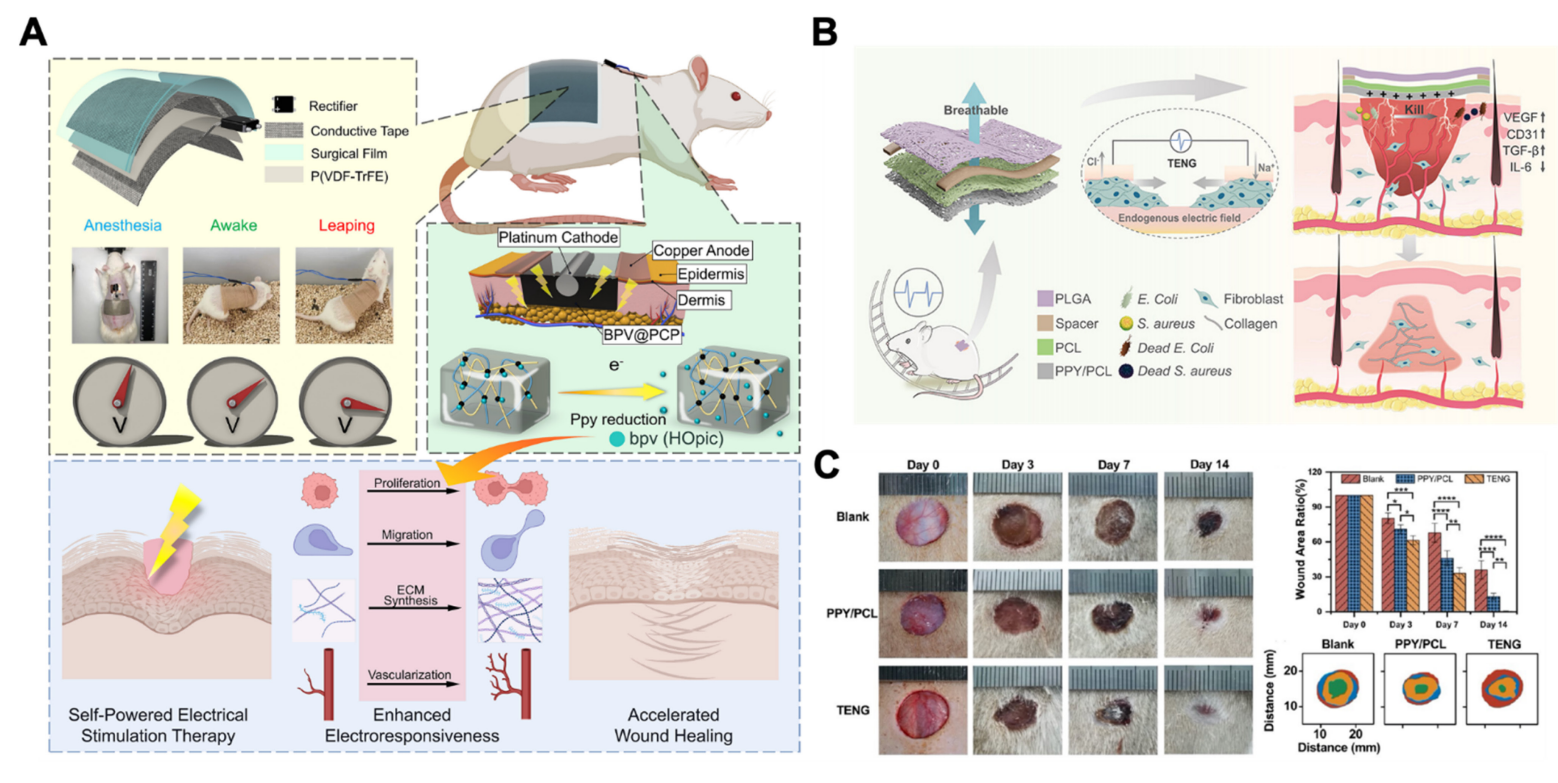
Electro-responsive nanomaterials for wound healing. A) Diagrammatic illustration of nanogenerator accelerates wound healing. Adapted with permission from 194. Copyright 2023, American Chemical Society. B) Diagrammatic illustration of triboelectric nanogenerator accelerates diabetic infected wound healing. C) Effect of TENG patch on diabetic infected wound healing in different treatments. Adapted with permission from 196. Copyright 2023, Elsevier.

Finally, combining piezoelectric nanomaterials with other therapeutic approaches can synergistically promote wound healing. First, piezoelectric nanomaterials can be combined with drug delivery. For example, phosphatase and tensin homolog (PTEN) has been proved to suppress cellular responsiveness to electrical stimuli **(Figure 12A)** 193. Therefore, combining piezoelectric nanomaterials with BPV, an inhibitor of PTEN, can amplify the cellular response to electrical stimulation. On this basis, researchers have developed a piezoelectric nanoplatform loaded with BPV (BPV@PCP). This nanoplatform can respond to physiological activities and convert them into electrical stimuli to control BPV release, thereby enhancing cell responsiveness to electrical stimulation. Compared with piezoelectric nanomaterials without BPV, this material effectively accelerates wound healing 194. Second, combining piezoelectric nanomaterials with cell membrane coating technology can also increase their efficacy. For instance, the use of macrophage membranes preactivated by *Staphylococcus aureus* to coat piezoelectric nanomaterials can increase their bactericidal activity. Compared with conventional macrophage membranes, piezoelectric nanomaterials preactivated by *Staphylococcus aureus* express higher levels of pathogen-associated molecular patterns (PAMPs), thereby enabling more precise bacterial recognition. Therefore, this piezoelectric nanomaterial can target infected areas and precisely exert bactericidal effects, thereby promoting wound healing. In addition to the above two approaches, other methods, such as modifying the surface charge properties and hydrophilicity/hydrophobicity of piezoelectric nanomaterials, can also enhance the interaction between them and bacteria or cells, thereby improving their efficacy in promoting wound healing 61.

#### 4.2.2. Triboelectric nanomaterials

**Figure 13:**
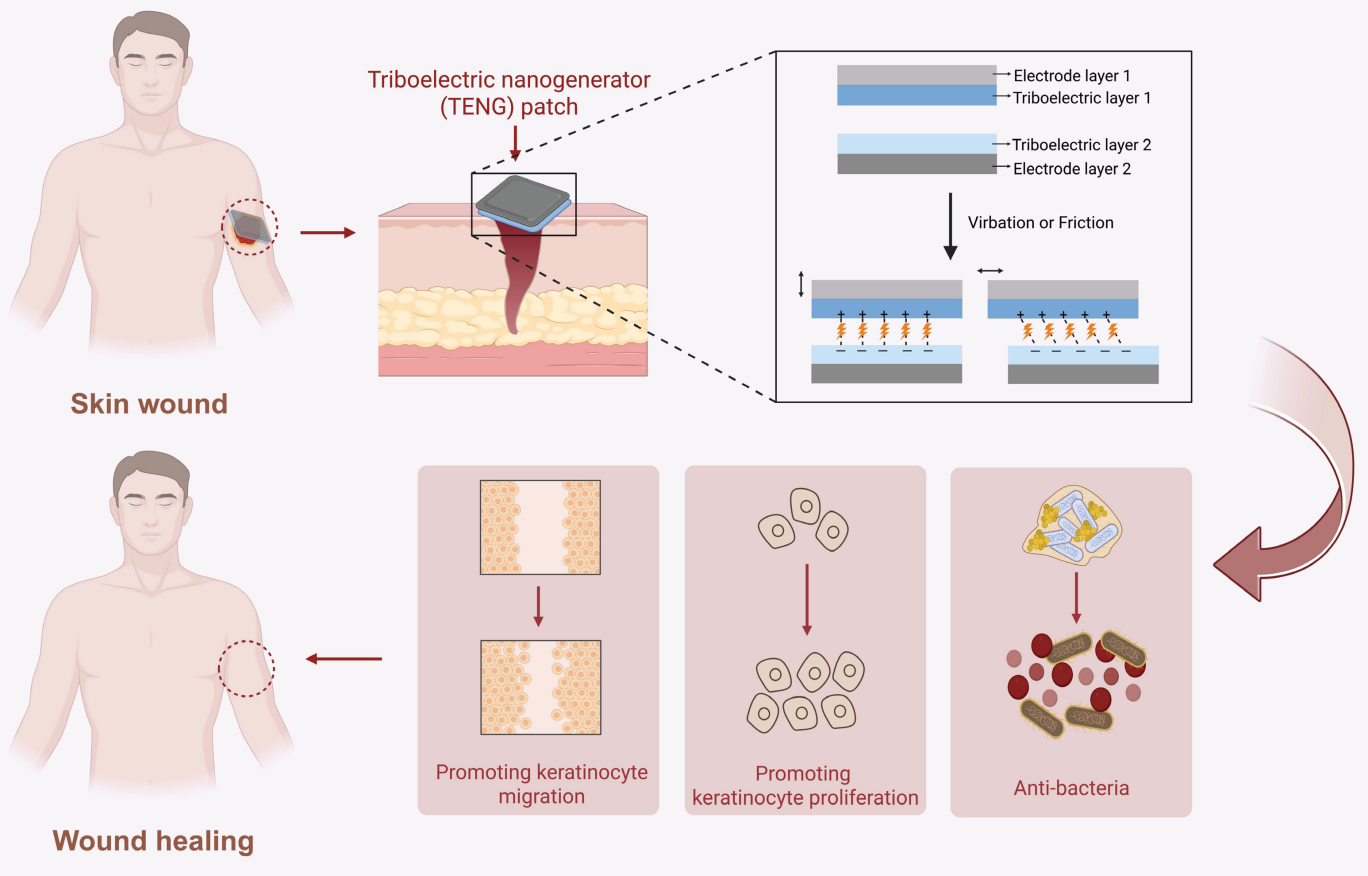
Diagrammatic illustration of the triboelectric nanomaterials applied in wound treatment. The figure shows how triboelectric nanomaterials convert mechanical friction into electrical signals and thereby promoting wound healing. Created in https://BioRender.com.

Triboelectric nanomaterials are mainly used in the form of triboelectric nanogenerators (TENGs), which consist of electrode layers and triboelectric layers with opposite charges. They primarily utilize the principles of electrostatic induction and contact electrification to convert mechanical force into electrical energy. TENGs mainly accelerate wound healing by exerting bactericidal effects and promoting cell proliferation and migration **(Figure 13)**.

Firstly, TENGs can exert bactericidal effect by mediating transient electroporation to disrupt bacterial membranes and promote H_2_O_2_ formation, thereby accelerating wound healing 180,195,196. For instance, researchers constructed a single-electrode TENG composed of Polypyrrole/ Polycaprolactone (PPY/PCL), PCL, and poly(lactic-co-glycolic acid) (PLGA) layers. Under physiological movement stimulation, two tribo layers (PCL and PLGA) vibrate according to the “contacted-separated-contacted” cycles. Because of their different electron affinities, electrons will transfer and cause regular changes in the open-circuit voltage and the short-circuit current. By the time TENG contacts with infected wound, the positively charged surface of TENG can effectively adsorb bacteria through electrostatic induction. Simultaneously, the electrical stimulation produced by TENG can produce H_2_O_2_ and induce electroporation of bacterial membranes, resulting in bacterial contents leaking, which achieves precise bactericidal effects and accelerates diabetic infected wound healing **(Figure 12B-C)** 196.

Secondly, TENGs can generate electric signals to regulate cellular functions, thereby promoting wound healing 197-200. For example, a TENG device (Electro-Generating Dressing, EGD) combined with negative pressure wound therapy (NPWT) can promote wound healing through regulating immune responses and accelerating the re-epithelialization process. Specifically, negative pressure therapy can induce the periodic contact-separation cycles within the TENG’s friction layer. Based on the different electron affinities of two tribo layers, the mechanical deformation can be converted into a stable electric field. On the one hand, this electric field can promote macrophages to polarize towards M2 phenotype and mitigate inflammation. On the other hand, it can activate the PI3K/Akt and MAPK/ERK signaling pathways to enhance the directional migration and proliferation of epidermal cells 197.

When electro-responsive nanomaterials are used to treat wounds, physical wear, wound exudate corrosion, and mechanical fatigue may gradually decrease their effectiveness 201. To address this issue, there are three ways to optimize material design. First, electro-responsive nanomaterials resistant to mechanical fatigue can be selected as the core components. Second, biocompatible coatings (such as polyethylene glycol) can be used to modify material surfaces, thereby protecting against the corrosive effects of wound exudate. Third, sensors can be integrated within the electro-responsive nanomaterials, and wireless communication technology can be integrated to monitor the material’s electrical output performance and wound healing status in real time, which can enable precise indication of the material replacement time, ensuring its treatment efficiency.

Most electro-responsive nanomaterials currently in use tend to overemphasize therapeutic efficacy while neglecting real-time feedback on wound status. This limitation may lead to issues such as the ability to monitor material effectiveness decreasing in real time (such as reduced electrical signal generation efficiency), which can cause treatment failure. Moreover, it is difficult to precisely adjust the electrostimulation intensity on the basis of the wound status, potentially leading to either excessive stimulation or insufficient efficacy. To overcome this challenge, in the future, electro-responsive nanomaterials can be integrated with wireless communication technology and artificial intelligence algorithms, thereby establishing a closed-loop therapeutic system featuring “sensing-analysis-regulation”. This system would rely on sensors to capture real-time signals of the electrical properties of the material and the wound microenvironment (such as pH and temperature). After analysis by AI algorithms, it would dynamically optimize electrical stimulation parameters, ultimately achieving more precise and adaptive wound interventions.

### 4.3. Magnetic-responsive nanomaterials

**Figure 14:**
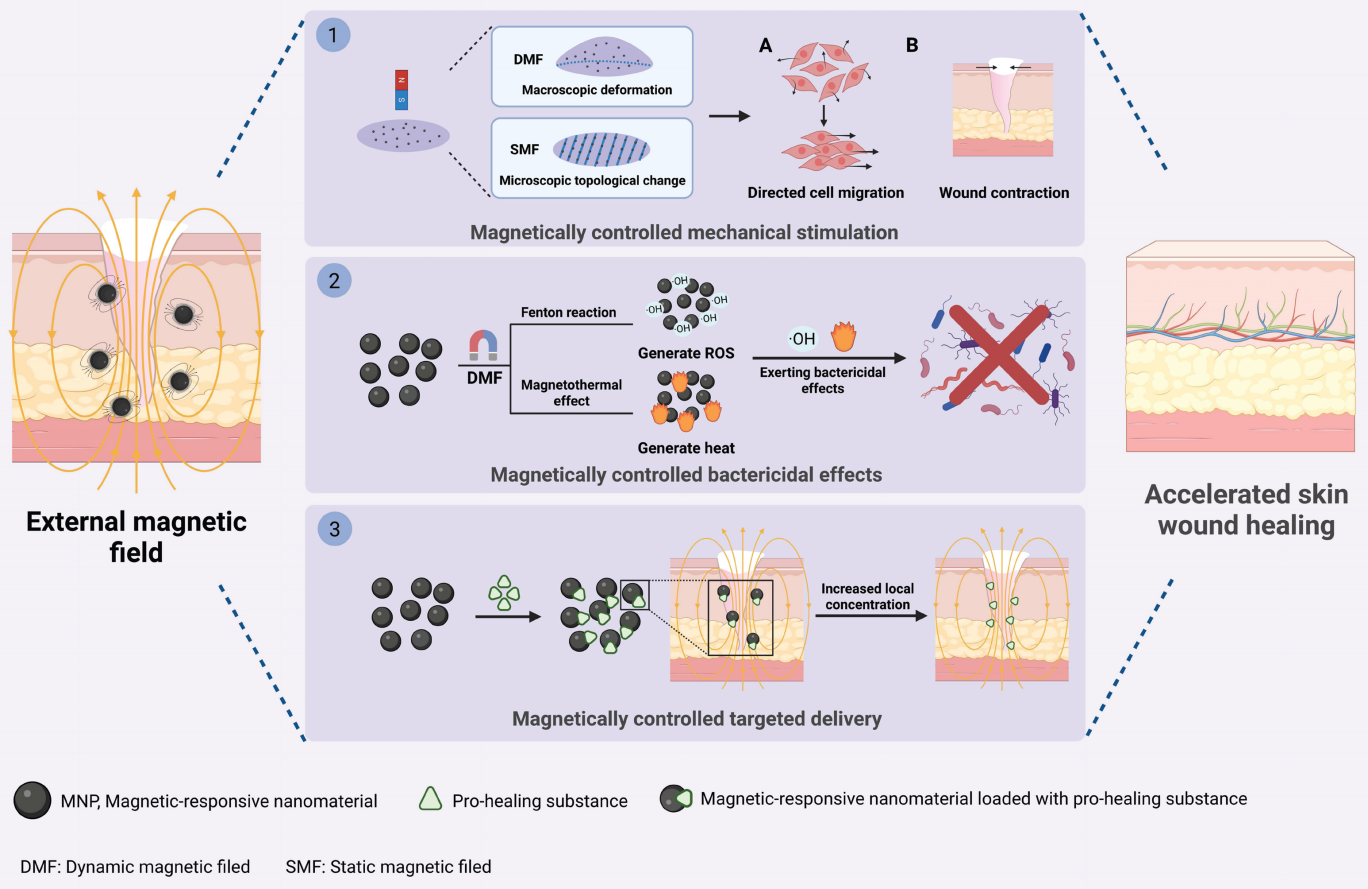
Diagrammatic illustration of the magnetic-responsive nanomaterials applied in wound treatment. The figure shows how magnetic nanomaterials (e.g., iron oxide nanoparticles) respond to an external magnetic field to accelerate wound healing. Upon magnetic field application, these nanomaterials can exert mechanical forces on skin tissue cells, generate local heat, produce ROS, or achieve targeted release of therapeutic agents, thereby promoting wound healing. Created in https://BioRender.com.

Magnetic-responsive nanomaterials refer to nanomaterials that exhibit specific physical or chemical responses to stimulation by an external magnetic field 202. They can accelerate wound healing through three mechanisms: (1) providing mechanical cues to skin tissue cells; (2) exerting bactericidal effects; and (3) enhancing the targeting ability of pro-healing substances **(Figure 14)**.

**Figure 15:**
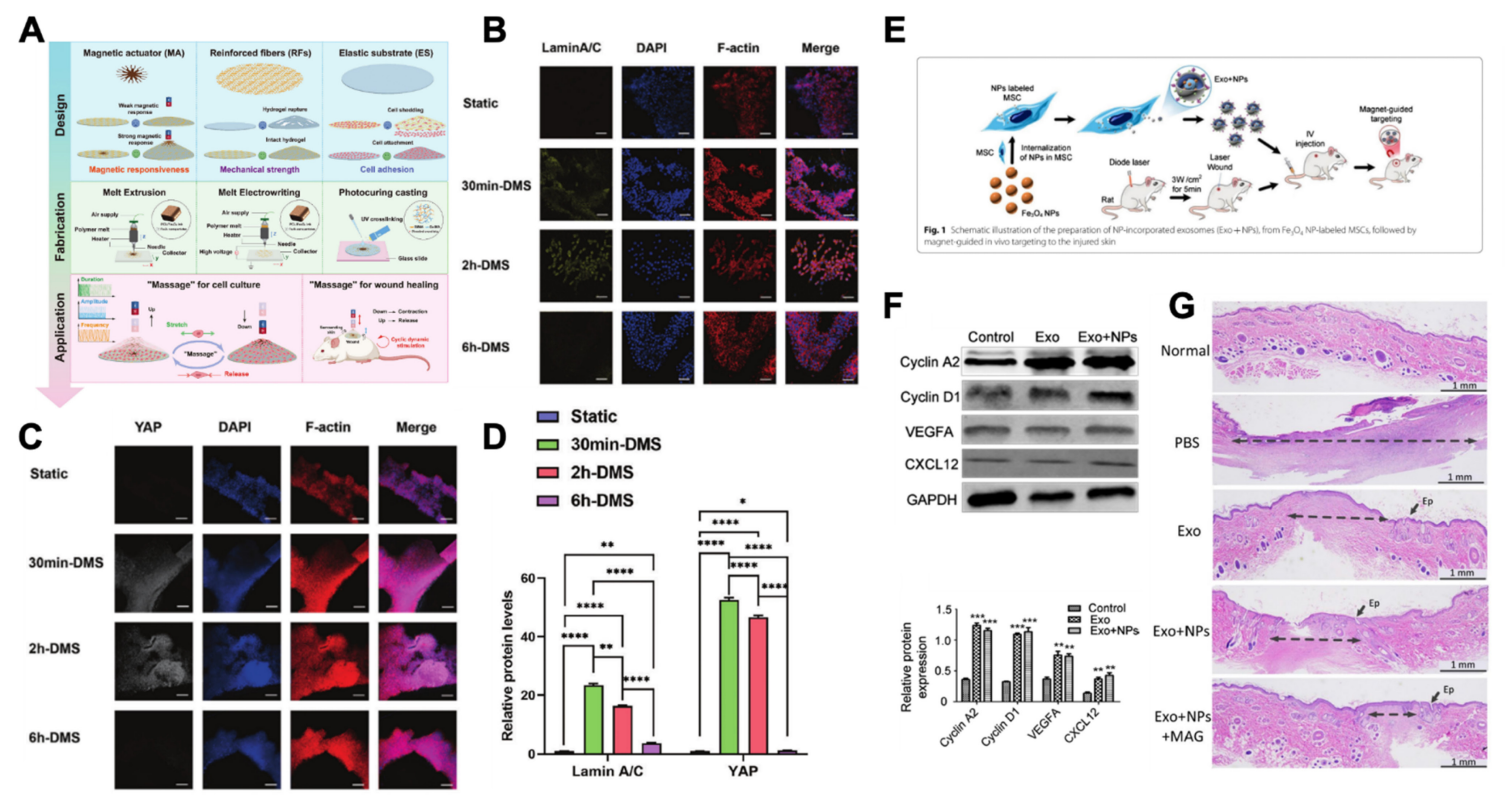
Magnetic-responsive nanomaterials for wound treatment. A) Diagrammatic illustration of the magnetic-responsive massage membrane accelerates wound healing. B) Effect of magnetic field duration on the expression level of mechanical transduction-related protein LaminA/C. C) Effect of magnetic field duration on the expression level of mechanical transduction-related protein YAP D) Relative expression level of two proteins under different magnetic field duration. Adapted with permission from 46. Copyright 2024, John Wiley and Sons. E) Description of the synthesis of nanoparticle-incorporated exosomes (Exo + nanoparticles) and its pro-healing effect in injured skin. F) Expression level of proteins associated with HUVEC proliferation, migration, and angiogenesis with different treatment. G) H&E staining of wound sections under different treatment with magnetic guidance 5 weeks post-wounding. Arrows indicate scar edges. Adapted with permission from 64. Copyright 2020, Springer Nature.

First, under static or dynamic magnetic field stimulation, magnetic-responsive nanomaterials can respond to and undergo macroscopic deformation or microscopic topological changes, thereby providing directional mechanical stimulation to skin tissue cells and promoting wound healing 46, 203-205. On the one hand, under the stimulation of external dynamic magnetic fields, membranes containing Fe_3_O_4_ nanoparticles will undergo periodic macroscopic deformation, which will activate mechanosensitive channels on fibroblast and keratinocyte membranes and upregulate the expression of the mechanosensitive proteins YAP and Lamin A/C. Consequently, this membrane can promote wound contraction and re-epithelialization to accelerate skin wound healing **(Figure 15A-D)** 46. On the other hand, under a static magnetic field, magnetic-responsive nanomaterials can undergo orientation according to the direction of the magnetic field, inducing changes in their microscopic topological structure and thereby promoting wound regeneration. For instance, under static magnetic field stimulation, magnetic-responsive cellulose nanocrystals within the Alg-SF-CNC composite nanoscaffold can align along the magnetic field direction, which will create an ordered topological structure within the scaffold, thereby providing migration guidance for fibroblasts and keratinocytes. Consequently, these cells migrate in an organized manner along the scaffold orientation to accelerate wound healing 204.

Second, magnetic-responsive nanomaterials can exert bactericidal effects under the influence of a magnetic field by generating ROS or through the magnetic heating effect, thereby accelerating the healing of infected wounds. For instance, AIron NPs can accelerate the release of Fe^2+^ in an alternating magnetic field, increase the amount of ·OH, and exert a bactericidal effect by oxidizing and destroying the bacterial cell membrane and nucleic acids, thereby promoting the healing of infected wounds. Additionally, under the stimulation of an alternating magnetic field, Fe_3_O_4_ NPs can convert magnetic energy into thermal energy and increase the local temperature at the wound to 50 °C, thereby exerting antibacterial and anti-inflammatory effects and accelerating the healing of diabetic infected wounds 206, 207.

Finally, magnetic fields, magnetic-responsive nanomaterials and pro-healing substances (such as growth factors, stem cells, and extracellular vesicles) can form a “magnetic targeting system” together 202. By modulating the magnetic field, the targeted accumulation of these substances at the wound site can be enhanced. This is because when the magnetic force exceeds the blood flow velocity, the pro-healing substances labeled with magnetic-responsive nanomaterials can be retained in the affected area, thereby increasing their local concentration 208. For example, in burn wound treatment, when magnetic-responsive Fe_3_O_4_ nanoparticles are internalized by MSCs, the secreted exosomes (MSC-Exos) are labeled with Fe_3_O_4_ nanoparticles. Under magnetic field stimulation, the homing of MSC-Exos to the wound site significantly increases, thereby rapidly promoting angiogenesis, collagen deposition, and re-epithelialization to accelerate burn wound healing **(Fig. 15E-G)** 64.

Currently, the “magnetic targeting system” still faces the challenge of limited depth of targeted delivery. This limitation is influenced mainly by the distance between the magnet and the magnetic-responsive nanomaterials, the blood flow at the wound site, and the circulation time of the nanoparticles.

The targeted aggregation effect of magnetic-responsive nanomaterials depends on the magnetic field strength, and the magnetic field decreases as the distance between the magnet and the magnetic-responsive nanomaterials increases 209, which results in magnetic-responsive nanomaterials having a targeted depth limited to less than 5 mm of the body surface 210. This restriction makes it difficult to meet the therapeutic component delivery requirements for deep wounds. Several possible strategies can be considered to overcome this limitation. First, single magnets can be replaced with array or multipole magnets to optimize the magnet architecture. This method mitigates the decrease in the magnetic field due to distance by using field superposition to increase the magnetic field strength and uniformity in deep wounds. Moreover, the sensitivity of magnetic-responsive nanomaterials to magnetic fields can be enhanced. The magnetic moment of magnetic-responsive nanomaterials can be increased by modification techniques such as adding metal dopants (including Zn, Co, and Mn), developing specific-shaped magnetic-responsive nanomaterials (such as cubic-shaped), and decreasing their size. These modifications will enable sufficient magnetic force even under distant weak magnetic fields, thereby achieving targeted aggregation. Through the above methods, the targeted effect of magnetic-responsive nanomaterials may be enhanced, making them more suitable for treating deep wounds.

Hemodynamics is also a key factor in determining whether magnetic nanomaterials can successfully remain at the wound site 211, 212. In the treatment of deep wounds, due to the weakened magnetic field gradient, the magnetic force often fails to fully resist the flushing effect caused by blood flow or a large amount of exudate, making it difficult for magnetic nanomaterials to stably remain in the wound area. To solve this problem, the surface of the nanoparticles can be modified with ligands (such as antibodies and peptides) that specifically bind to the surface of target cells in the target area 214. By utilizing the high affinity between the receptor and ligand, combined with magnetic force, magnetic nanomaterials can enhance their adhesion to the wound tissue, jointly resisting the shear forces of local blood flow and exudate, thereby facilitating the stable retention of the materials at deep wound sites.

Finally, the *in vivo* circulation time of magnetic nanomaterials is another factor for their effectiveness 215. The mononuclear phagocytic system is the main obstacle for rapid clearance of nanoparticles from the body. Researchers have designed various strategies to enable nanoparticles to evade recognition by the MPS, thereby prolonging their *in vivo* circulation time. These strategies include coating the surface of the nanoparticles with hydrophilic polymers (such as PEGylation) to form a hydration layer to inhibit plasma protein absorption 216; designing biomimetic nanoparticles (such as red blood cell membranes and platelet membranes) 217; and using specific cells (such as neutrophils and monocytes) as carriers to implement strategies such as “cell hitchhiking”. These strategies could extend the circulation half-life of the nanoparticles in the blood, providing the necessary circulation time basis for their targeted enrichment in deep tissues under the influence of the magnetic field 218.

### 4.4. Other exogenous stimuli-responsive nanomaterials

Besides the commonly used light-, electro-, and magnetic-responsive nanomaterials mentioned earlier, other exogenous stimuli-responsive (such as ultrasound and temperature) nanomaterials can also promote wound healing and have achieved significant research breakthroughs.

Ultrasound-responsive nanomaterials can accelerate wound healing through three mechanisms: (1) promoting nerve and vascular regeneration; (2) modulating immune responses and (3) exerting bactericidal effects.

First, ultrasound-responsive nanomaterials can accelerate wound healing by converting ultrasonic waves into electrical signals, thereby promoting nerve and vascular regeneration 67, 219. Under the stimulation of ultrasound waves, ultrasound-responsive nanomaterials with non-centrosymmetric structures can undergo internal charge separation between positive and negative charges, thereby generating an internal electric field and producing electrical signals. For example, hydrogels containing ultrasound-responsive nanoparticles (K,Na)NbO_3_ can convert ultrasonic waves into electrical signals to activate the AMPK pathway, increasing the transmembrane transport of nerve growth factor-containing vesicles by promoting ATP synthesis. Consequently, this nanohydrogel can enhance the neurotrophic factors secreted by Schwann cells (such as NGF, BDNF, and VEGF), thereby inducing nerve and vascular regeneration. This process enhances local oxygen levels and healing-related growth factor secretion to accelerate diabetic wound healing 67.

Second, ultrasound-responsive nanomaterials can accelerate wound healing by modulating immune response 66, 220. Under the effect of ultrasound, platinum nanoparticle assemblies (PNA) can promote electron polarization through the plasma resonance effect, thereby generating GSH to scavenge high concentrations of oxidative substances in the wound microenvironment. This subsequently accelerates diabetic wound healing by regulating immune responses and restoring the proliferation and migration capabilities of fibroblasts and keratinocytes 66.

Finally, ultrasound-responsive nanomaterials can promote wound healing by generating ROS to exert bactericidal effect 221, 222. Under the stimulation of ultrasound, electrons within ultrasound-responsive nanomaterials will undergo transitions. When these transitioned electrons return to their ground state, the released energy can be transferred to substances such as O_2_ to generate ROS 223. For example, under ultrasound stimulation, oxygen-rich vacancy porous titanium dioxide nanosheets/ultrafine copper peroxide nanoclusters (OV-TiO_2_/CuO_2_) can generate ROS to kill MRSA and suppress excessive inflammatory responses, thereby accelerating MRSA-infected wound healing 222. For another example, under ultrasound stimulation, the two-dimensional Bi_2_WO_6_ nanosheets can generate ROS, which induces lipid oxidation and membrane rupture of bacterial cell membranes, thereby exerting bactericidal and accelerating the healing of MRSA-infected wounds 224.

In response to temperature changes at the wound site, thermo-responsive nanomaterials can undergo structural transformation and drive early wound closure, thereby accelerating wound healing. During the healing process, wound temperature undergoes dynamic changes. During the inflammatory stage, the wound temperature exceeds the normal surface skin temperature 225,226. Nanomaterials can respond to this change and accelerate wound healing 68, 227, 228. For example, when wound temperature is above 37 °C, the polyurethane/polyvinyl butyral (PU/PVB) nanofiber membrane can release its stored energy, undergo centripetal contraction, and generate “micro-deforming forces” along the wound edges. This can increase fibroblast proliferation and migration, promote early wound closure and shield the healing process from external adverse factors (such as bacteria or foreign bodies), thereby providing a stable microenvironment that expedites wound healing 68.

However, there is currently limited application of temperature-responsive nanomaterials in wound treatment. This may be because their effect of accelerating wound closure only provides a physical barrier to the healing process, and their effective duration is relatively constrained. To enable temperature-responsive nanomaterials to more comprehensive effects in promoting wound healing, firstly, it is advisable to develop temperature-responsive nanomaterials with specific morphologies. For instance, nanofibers with porous structures, aligned structures, or directional orientation have been proven to provide directional guidance for the migration of skin tissue cells, thereby accelerating skin tissue regeneration. Secondly, it is recommended to combine temperature-responsive nanomaterials with antibiotics, growth factors, etc., thus promoting wound healing through multiple effects.

## 5. Dual stimuli-responsive nanomaterials

**Figure 16:**
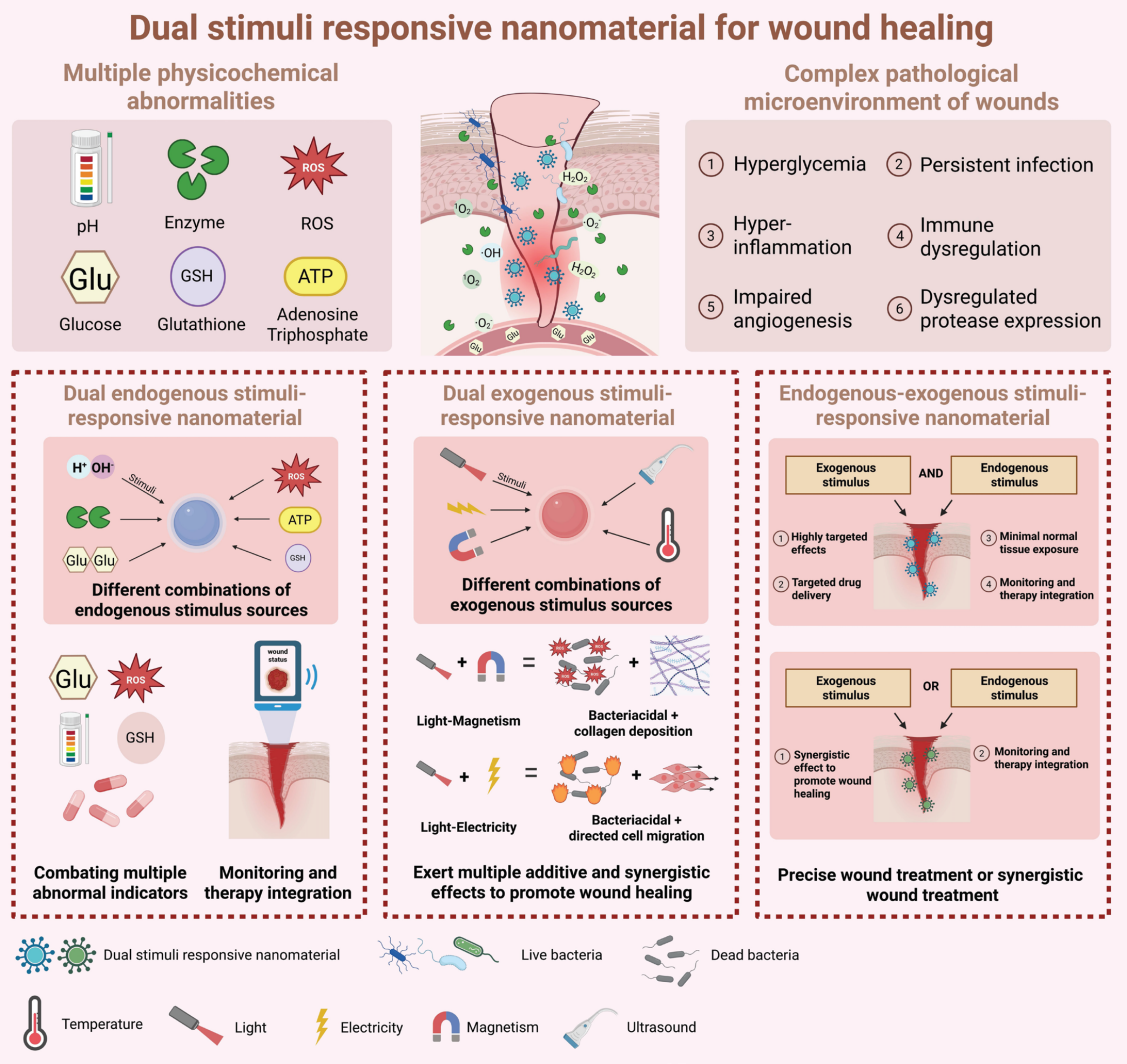
Diagrammatic illustration of dual stimuli-responsive nanomaterials applied in wound treatment. The figure shows how these nanomaterials are designed to respond to two complementary triggers. Upon sequential or simultaneous exposure to both stimuli, the nanomaterials undergo synergistic structural changes or energy conversion, leading to on-demand release of therapeutics or enhanced physical effects, thereby promoting wound healing. Created in https://BioRender.com.

The previous text has already elaborated on the role of single stimuli-responsive nanomaterials in wound treatments. To further enhance the functionality of stimuli-responsive nanomaterials, researchers have designed dual stimuli-responsive nanomaterials. They can respond to two distinct stimulus sources, thereby exerting multiple functions to promote wound healing. These mainly include (1) dual endogenous stimuli-responsive nanomaterials; (2) dual exogenous stimuli-responsive nanomaterials and (3) endogenous-exogenous stimuli-responsive nanomaterials **(Figure 16)**.

### 5.1. Dual endogenous stimuli-responsive nanomaterials

**Figure 17:**
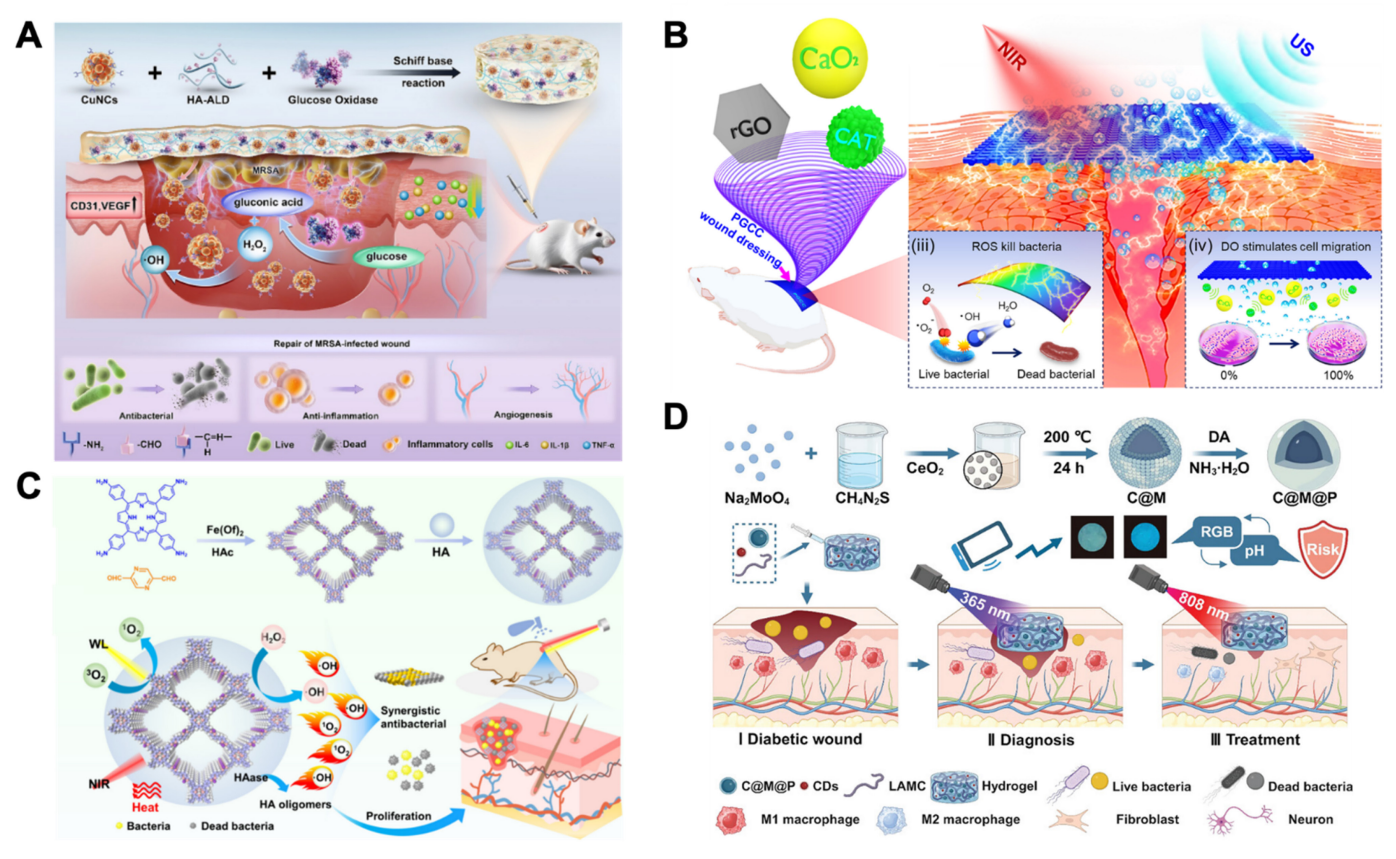
Dual stimuli-responsive nanomaterials for wound treatment. A) Diagrammatic illustration of CGH hydrogel for diabetic wound. Adapted with permission from 55. Copyright 2024, Elsevier. B) Diagrammatic illustration of PGCC for infected wound. Adapted with permission from 71. Copyright 2023, Elsevier. C) Diagrammatic illustration of the preparation process of HA-Fe-COF and its pro-healing effect for infected chronic wound. Adapted with permission from 72. Copyright 2024, American Chemical Society. D) Diagrammatic illustration of LAMC/CD-C@M@P hydrogel for diabetic wound. Adapted with permission from 237. Copyright 2024, John Wiley and Sons.

Dual endogenous stimuli-responsive nanomaterials refer to nanomaterials that can respond to two kinds of endogenous stimulus sources. For example, pH-glucose-responsive nanomaterials, pH-enzyme-responsive nanomaterials, and pH-GSH-responsive nanomaterials 55, 229, 230. Such stimuli-responsive nanomaterial can confront the complex wound microenvironment with multiple abnormal indicators, such as diabetic wounds and bacterially infected wounds. Besides, they can also monitor wound physicochemical indicators during wound therapy. For instance, researchers designed glucose-pH-responsive nanohydrogel copper nanoclusters/GOx/oxidized hyaluronic acid (CGH) to deal with the hyperglycemic and acidic pH microenvironment of diabetic wounds. Firstly, the GOx incorporated in the nanohydrogel can reduce glucose levels. Subsequently, the acidic pH at the wound site triggers the release of copper nanoclusters (CuNCs), which then exert bactericidal and anti-inflammatory effects through the Fenton reaction. This nanohydrogel can promote re-epithelialization, collagen deposition, and vascular regeneration to accelerate diabetic wound healing **(Figure 17A)** 55.

### 5.2. Dual exogenous stimuli-responsive nanomaterials

Dual exogenous stimuli-responsive nanomaterials refer to nanomaterials that can respond to two kinds of exogenous stimulus sources. Such as light-electro and light-magnetic-responsive nanomaterials. This type of stimuli-responsive nanomaterial can synergistically promote wound healing through combined effects 71, 231-233. For instance, light-electro-responsive nanomaterials (PGCC) containing poly-L-lactic acid (PLLA), reduced graphene oxide (rGO), calcium peroxide (CaO_2_) and CAT that exert photothermal and piezoelectric effects can accelerate infected wound healing by exerting bactericidal effect and promoting tissue regeneration. Under ultrasound stimulation, PLLA generates oxidative substances through piezoelectric effect to exert bactericidal and anti-inflammatory effects. Meanwhile, under 808 nm near-infrared laser irradiation, rGO can facilitate the photothermal effect and produce mild thermal stimulus, thereby up-regulating the secretion of heat shock protein 90 (Hsp90) to maintain protein homeostasis and accelerate tissue regeneration **(Figure 17B)** 71. In addition, by employing a “dual-drive” strategy, researchers developed a magnetic nanoparticle-protein fiber complex (GMPF_0.1_) with oriented fibrillar structures containing magnetic nanoparticles and coated with polydopamine (PDA). Specifically, under 808 nm near-infrared laser irradiation, the PDA shell generates photothermal effects and produces thermal stimulation to disrupt bacterial cell membranes, thereby achieving bactericidal effect. Subsequently, magnetic nanoparticles undergo directional alignment under magnetic field stimulation, further inducing the formation of highly oriented fiber structures within the magnetic nanoparticle-protein fiber complex. This oriented structure can provide growth guidance for fibroblasts and regulates their ordered proliferation and migration, thereby accelerating tissue regeneration and ultimately promoting bacterial-infected wound healing 233.

### 5.3. Endogenous-exogenous stimuli-responsive nanomaterials

Endogenous-exogenous stimuli-responsive nanomaterials refer to nanomaterials that can respond endogenous and exogenous stimulus. Such as pH-light-responsive, ROS-light-responsive, enzyme-light-responsive, and pH-electro-responsive nanomaterials 72, 173, 234-236. The characteristics of such nanomaterials are as follows: Firstly, they can initiate a response triggered by endogenous stimuli within the wound microenvironment, while simultaneously combining with exogenous stimuli to enhance therapeutic efficacy. Secondly, these nanomaterials can monitor wound physicochemical indicators during wound therapy enabled by endogenous stimuli, enabling real-time assessment of wound state and achieving integrated “monitoring-treatment” capabilities. For instance, researchers designed an enzyme-light-responsive HA-Fe-COF nanoplatform to promote bacterially infected wound healing. Only when the hyaluronidase secreted by bacteria degrades the HA encapsulated on the surface of the nanoplatform, Fe-COF can be released. Subsequently, under the irradiation of NIR light, it can generate the PTDT effect to exert advanced bactericidal effect, thereby ensuring that the material only functions in the presence of bacteria, improving antibacterial efficiency, and preventing the generation of heat and ROS from damaging the surrounding normal skin tissue **(Figure 17C)** 72. Additionally, by incorporating ceria-molybdenum disulfide nanoparticles (C@M@P) and carbon quantum dots (CDs) encapsulated in a polydopamine shell into a hydrogel, researchers developed a pH-light-responsive hydrogel that can both monitor and treat diabetic infected wounds. First, CDs exhibit photoluminescence properties. Specifically, when excited by light at specific wavelengths, they exhibit pH-sensitive fluorescence emission based on protonation/deprotonation reactions of surface functional groups and defect-state electron transitions. Consequently, under UV irradiation, the fluorescence intensity emitted by CDs linearly increases with rising pH values. Based on this property, fluorescent images of the hydrogel can be captured by smartphone and converted into RGB signals to rapidly estimate wound pH levels, enabling early warning of bacterial infections (typically an alkaline pH environment). When monitored pH signals indicate bacterial infection, under 808 nm near-infrared irradiation, C@M@P can generate high-temperature thermal stimulation through photothermal effects to exert bactericidal effects. Simultaneously, the triggered photothermal effects can also accelerate the valence transition of Ce^3+^ to Ce^4+^ in cerium dioxide through thermal energy input. This enhances C@M@P’s ROS scavenging capacity, ultimately accelerating diabetic wound healing through synergistic bactericidal, anti-inflammatory, and antioxidant effects **(Figure 17D)** 237.

## 6. Types of wound applied with stimuli-responsive nanomaterials

### 6.1. Acute wound healing

Acute wounds are usually caused by accidental injuries or cuts and generally heal within a few weeks. They are characterized by rapid injury occurrence, fast repair processes, and relatively simple healing. The core goal is to prevent infection and achieve rapid closure of the wound 3, 238. On the basis of the acidic pH characteristics during the inflammatory stage of acute wounds, acidic pH-responsive nanomaterials can release Ag⁺ in a low-pH environment, exerting bactericidal effects and accelerating the healing of acute wounds 239. Currently, the application of stimuli-responsive nanomaterials for acute wounds is relatively limited. On the basis of these findings, temperature-responsive, electro-responsive, and magnetic-responsive nanomaterials can promote the migration of fibroblasts and keratinocytes and accelerate wound contraction; therefore, these materials may have considerable application prospects for acute wounds.

### 6.2. Chronic wound healing

#### 6.2.1. Chronic infected wound healing

When a wound becomes infected, bacteria colonize the wound site and trigger an uncontrolled inflammatory response, resulting in delayed healing. The pathological microenvironment of infected wounds is characterized by the accumulation of bacterial metabolites, pH imbalance, and elevated oxidative stress levels 240, 241. For these wounds, pH, enzymes, ROS, and light- and magnetic-responsive nanomaterials can all have therapeutic effects, but the choice of treatment depends on the pathological characteristics.

In infected wounds where biofilms have not yet formed or where only a thin layer of biofilm exists, the acidic pH and elevated ROS microenvironment can enable pH- and ROS-responsive nanomaterials to exert antibacterial and anti-inflammatory effects through drug delivery or mimicking enzyme activity, thereby accelerating wound healing 242, 243. This is because the dense extracellular polymer (EPS) matrix of the biofilm can hinder the penetration of pH- and ROS-responsive nanomaterials, making it difficult for them to act on bacteria deep within the biofilm 244. Additionally, for wounds where specific bacteria need to be eliminated and the biofilm has not yet fully formed, enzyme-responsive nanomaterials, which incorporate chemical bonds recognized by specific bacterial enzymes (such as the V8 protease secreted by *Staphylococcus aureus*), are specifically degraded by these enzymes at the site of infection, thereby precisely releasing antimicrobial drugs. This enables the selective elimination of target pathogens and accelerates wound healing 245.

In infected wounds where biofilms have formed, light-responsive nanomaterials can generate local high-temperature or reactive oxygen species under external light irradiation, effectively destroying the EPS matrix of the biofilm and promoting the penetration of the material into the deeper layers of the biofilm to exert bactericidal effects 246. Magnetic-responsive nanomaterials are suitable for infected wounds with deep biofilm colonization because of their advantages, such as strong tissue penetration force and noninvasive and controllable characteristics. They can rapidly lyse the biofilm under the influence of a magnetic field, effectively eliminating internal bacteria and accelerating wound healing 247.

#### 6.2.2. Chronic diabetic wound healing

In diabetic wounds without bacterial infection, the main pathological features include hyperglycemia, elevated levels of oxidative stress, excessive degradation of the extracellular matrix (ECM), and impaired angiogenesis 107. In response to these pathological features, various stimuli-responsive nanomaterials can exert corresponding therapeutic effects. Specifically, in the alkaline microenvironment of such wounds, pH-responsive nanomaterials can respond to the alkaline environment by releasing therapeutic substances or exerting nanoenzymatic activity, thereby accelerating wound healing. In response to excessive ECM degradation and poor formation of granulation tissue, the expression levels of specific host enzymes (such as MMPs and elastase) in the local microenvironment are relatively high. At this time, enzyme-responsive nanomaterials can exert therapeutic effects through drug delivery 51. In terms of the characteristics of persistent inflammation and high oxidative stress levels, ROS-responsive nanomaterials can respond to ROS in the microenvironment and exert therapeutic effects by releasing bioactive substances and eliminating excessive ROS 52. In response to the significant fluctuations in glucose concentration in the wound microenvironment, glucose-responsive nanomaterials can specifically respond to the high-glucose microenvironment of the wound and break down glucose to accelerate wound healing 55. In terms of the poor formation of microenvironmental blood vessels and the extracellular matrix, nanomaterials that can generate mild-temperature photothermal effects can promote granulation tissue regeneration and re-epithelialization 248, and magnetic-responsive nanomaterials can also play a similar role by generating mechanical stimulation to accelerate tissue repair 249. In addition, in terms of persistent oxidative stress and poor vascular formation, electro-responsive nanomaterials can simulate the activity of natural enzymes, generate electrical signals, eliminate excessive ROS, and accelerate tissue regeneration 67.

With respect to diabetic wounds with bacterial infection, in addition to the abovementioned microenvironmental characteristics of simple diabetic wounds, infections in this type of wound often persist and are prone to forming biofilms. For such complex wounds, all kinds of stimuli-responsive nanomaterials have good therapeutic effects. On the basis of the above pathological characteristics, light-responsive nanomaterials that generate high-temperature photothermal effects and magnetic-responsive nanomaterials can not only exert rapid and efficient bactericidal effects but also destroy bacterial biofilms under the action of external physical fields, thereby effectively accelerating the healing of such wounds 58, 250. Furthermore, glucose-responsive nanomaterials can respond to the high-glucose environment of wounds, break down glucose and produce ROS, working together to lower blood sugar levels and kill bacteria, thereby accelerating wound healing 115.

#### 6.2.3. Chronic burn wound healing

The pathological characteristics of the burn wound microenvironment primarily include an excessive inflammatory response, high levels of oxidative stress, susceptibility to infection, and impaired angiogenesis 3. pH, ROS, enzymes, light, and electro-responsive nanomaterials all have great application prospects in burn wounds, but must be reasonably selected on the basis of the pathological characteristics of the wounds.

Changes in the pH of burn wounds are complex. Initially, wounds are alkaline, and as infection or inflammation progresses, they can become acidic, accompanied by high levels of ROS 251. At this time, pH-responsive and ROS-responsive nanomaterials can accelerate wound healing by delivering drugs and removing excessive amounts of ROS 252, 253. In terms of poor vascular regeneration, electro-responsive nanomaterials can accelerate wound healing by accelerating vascular regeneration and collagen fiber deposition 253. Because burn wounds are prone to infection, light-responsive nanomaterials can exert bactericidal effects through high-temperature photothermal effects, photodynamic effects and PTDT effects to accelerate wound healing 254.

## 7. Conclusion and outlook

Despite their significant application potential in promoting skin wound healing, stimuli-responsive nanomaterials still face several challenges that urgently require resolution. In each section, we have identified the specific challenges faced by different types of these nanomaterials in wound healing applications. Below, we will systematically summarize these challenges and discuss future research directions.

### 7.1. Retention capability and adaptability

Stimuli-responsive nanomaterials exhibit general challenges in terms of their wound retention ability and adaptability to exudative environments. In wounds with abundant exudate, stimuli-responsive nanomaterials can hardly maintain stable adhesion to the wound surface and are easily removed by exudate, resulting in a short effective duration and hindering long-term therapeutic effects. To overcome this problem, future studies should concentrate on developing *in situ* curing and active anchoring stimuli-responsive nanosystems. On the one hand, *in situ* gelation or self-assembling nanomaterials can form a stable three-dimensional network structure when in contact with wound exudate or specific enzyme environments, thereby physically blocking the flushing of wound exudate. On the other hand, dynamic covalent chemistry can be utilized to engineer the surface of the materials; for instance, modifying stimuli-responsive nanomaterials with phenylboronic acid groups can enable the formation of reversible covalent bonds between the abundant salivary acid residues in the skin tissue and the materials, resulting in an increase from “passive adhesion” to “active anchoring”, thereby significantly prolonging the retention time and treatment window of the stimuli-responsive nanomaterials at the wound site.

### 7.2. Protein corona effect

Upon contact with wounds, endogenous stimuli-responsive nanomaterials can rapidly adsorb proteins from wound exudate to form protein coronas. These coronas primarily consist of opsonin (such as coagulation proteins and complement proteins) and anti-opsonin (such as apolipoproteins and albumin). Because of their high specific surface area and irregular morphology, endogenous stimuli-responsive nanomaterials readily adsorb opsonin-based protein coronas. The formation of such protein coronas obscures the response sites on the material surface that perceive and respond to microenvironmental stimuli, preventing the release of therapeutic components; moreover, it can alter the inherent physicochemical properties of the material, compromising its “stealth” characteristics and thereby mediating the recognition of immune molecules such as complement proteins as signaling molecules by immune cells, prompting the material to be identified as a foreign object and subsequently phagocytosed and cleared. Consequently, this significantly decreases the circulation and functional time of endogenous stimulus-responsive nanomaterials within the wound site. Several possible strategies can be considered to overcome this limitation, such as suppressing conformation-regulating protein adsorption. Grafting polyethylene glycol or zwitterionic polymers onto the surface of stimuli-responsive nanomaterials can result in the formation of a dense hydration layer, thereby reducing protein binding through steric hindrance and electrostatic repulsion; alternatively, the construction of nanoscale topological structures (e.g., nanocolumns) on the stimuli-responsive nanomaterial surface to minimize the protein-surface contact area can also lower the adsorption energy and thereby suppress conformation-regulating protein adsorption.

### 7.3. Disordered response sequence

Although dual-stimuli-responsive nanomaterials display promising application potential in complex wound environments because of their ability to integrate the ability to perceive both endogenous and exogenous signals, it is worth noting that most current dual-stimuli-responsive nanomaterials possess only the physical capacity to respond to dual stimuli but lack intrinsic logical control mechanisms. This lack of control results in the risk of mutual interference between functional modules. When exposed to multiple stimulus signals, dual stimuli-responsive nanomaterials may experience competing or complex response pathways, leading to uncontrolled drug release and therapeutic effects. To achieve precise regulation and orderly activation of dual-response units, future studies could concentrate on the following two directions.

The first solution is to develop a spatiotemporal decoupled response mechanism. Through precise structural design, the physical isolation of different response modules of stimuli-responsive nanomaterials in space and sequential activation in time can be achieved. For example, in a light- and pH-responsive nanosystem, placing the light-sensitive unit and the pH-responsive unit at different spatial levels of the core-shell structure could prevent functional interference.

The second solution is to design dual stimuli-responsive nanomaterials on the basis of the NOT, YES, OR, and AND logic gates of Boolean logic operations in computer science. For example, “AND”-type dual stimuli-responsive nanomaterials, which must simultaneously meet the condition restrictions of two independent parameters, should be developed to ensure that the materials respond only under specific conditions, thereby significantly improving the accuracy and spatiotemporal specificity of wound treatment.

Currently, Boolean logic gates include mainly DNA logic gates and logic gates constructed on the basis of enzyme cascade reactions. The former relies on the specific recognition of DNA molecules to guide the reaction pathways and sequences, thereby generating corresponding logical functions. However, its application is primarily limited by the following factors: DNA degradation by nucleases in physiological environments, which causes logic gates to fail; potential immunogenicity; signal leakage; and complex synthesis methods. The latter uses the substrates and products of enzymes as input and output signals and performs logical operations such as AND, OR, NOT, and XOR through the design of single-enzyme catalytic reactions or multienzyme cascade reactions. However, its stability is easily disrupted by the microenvironment of the wound (such as temperature and pH), and there are problems such as logical errors caused by cross-reactions during application, difficulty in system reset caused by irreversible enzymatic reactions, and poor biocompatibility. Due to the high heterogeneity of the wound microenvironment and the incomplete evaluation system for the biocompatibility of related nanomaterials, the transformation and application of this stimuli-responsive platform based on Boolean logic gates in the field of wound healing still face significant challenges. Therefore, in the future, more in-depth research should be conducted on the application of this nanotechnology platform in skin wound healing to accelerate its development and transformation.

### 7.4. AI assisted material design

Due to the diversity and complexity of pathological wound microenvironments, traditional methods struggle to rapidly screen and design stimulus sources and nanomaterial combinations that match specific pathological microenvironments. Future applications of AI, with its unique modeling and predictive capabilities, may resolve this challenge. For instance, after the wound type and specific requirements for AI are described, vast datasets can be analyzed to recommend suitable stimulus sources, nanomaterials, and their combination methods. Furthermore, AI can also establish calculation models that predict variables such as a nanomaterial’s reaction time to particular stimuli, which can help determine the best nanomaterial ratios, combinations, and structures for wound healing. Additionally, dual stimuli-responsive nanomaterials remain the main focus of wound healing applications. However, in intricate wound microenvironments with several aberrant indicators, it is difficult for these materials to meet therapeutic requirements. Thus, AI-guided development of multistimuli-responsive nanomaterials should be the focus of future research. AI can suggest multistimulus combination techniques by examining the ways in which nanomaterials respond to various stimuli and their interactions. Furthermore, AI can intelligently control the order of responses to different stimuli after creates predictive models and simulate the reactions of nanomaterials to multiple stimuli. This enables the precise design of multistimuli-responsive nanomaterials with optimal performance. Consequently, this approach reduces trial-and-error costs and advances materials toward “intelligent adaptation”. This finding further supports the development goals of intelligent and precision-driven clinical treatment.

### 7.5. Biocompatibility

Cytotoxicity is the basis for evaluating the *in vitro* biocompatibility of materials. Ideal stimuli-responsive nanomaterials should not only exert therapeutic effects but also avoid damaging the surrounding tissue cells of the wound, thereby achieving more effective biological effects for wound healing. However, some stimuli-responsive nanomaterials still have potential cytotoxic effects. For instance, some nanoenzymes that can respond to endogenous stimuli have poor degradation performance because of their metal components and may have potential cytotoxicity. To avoid this issue, it is advisable to introduce biocompatible natural polymer materials or synthetic biodegradable polymers into the material system to enhance the biological safety of such materials.

In terms of *in vivo* biological safety, when stimuli-responsive nanomaterials enter the circulation system, they can be recognized by the immune system, leading to immune activation, immune suppression or immune disorders. Existing studies have not yet systematically clarified the influence of the physicochemical properties of nanomaterials on the immune response of the body. Moreover, most studies focus only on the induction of immune responses in the wound area by stimuli-responsive nanomaterials and their therapeutic effects and lack a comprehensive assessment of the systemic immune effects and potential negative effects they induce. Therefore, future research should focus on the precise regulation of the material’s physicochemical properties and the resulting immune responses and systematically evaluate the systemic immune responses it triggers. While maintaining therapeutic efficacy, the material should reduce nonspecific immune toxicity and achieve a balance between immune safety and efficacy. In addition, degradation characteristics and long-term biocompatibility are key factors affecting the *in vivo* biocompatibility of stimuli-responsive nanomaterials. When these materials enter the body, they can be internalized by the mononuclear phagocytic system and accumulate in organs such as the liver and spleen. Low degradability will lead to long-term material deposition, causing chronic inflammation and tissue damage through the induction of oxidative stress, inflammatory activation, and immune responses. However, current research focuses mostly on short-term biocompatibility assessments of stimuli-responsive nanomaterials and neglects long-term safety. Therefore, future design should prioritize the degradability of the materials and strengthen the systematic assessment of degradation products and long-term *in vivo* biocompatibility.

### 7.6. Regulation and scalability

In terms of regulatory approval, due to characteristics such as requiring external stimulation and ability to load bioactive substances, stimuli-responsive nanomaterials often possess multiple attributes, including drugs, biological products, and medical devices, resulting in ambiguous regulatory attributes and making it difficult for researchers to clearly identify the applicable laws and regulations. These multiple attributes also mean that the material must simultaneously meet the regulatory requirements for different products, significantly prolonging the research and clinical translation cycles. Moreover, there is currently a lack of efficacy and safety standards for such materials, restricting cross-study comparisons. Therefore, in the future, their regulatory attributes should be strictly defined, and unified standards should be established to accelerate clinical transformation.

In terms of large-scale production, the complex pathological microenvironment of wounds poses high requirements for the design and fabrication of stimuli-responsive materials. Researchers need to precisely control the physical and chemical parameters of materials to achieve precise drug release or a continuous response to multiple stimuli. Small-scale stable synthesis under laboratory conditions can be achieved. However, in industrial-scale large-scale production, process scaling often leads to unstable product efficacy and inconsistent quality between batches. In the future, advanced technologies such as microfluidic continuous flow synthesis technology and ultrasonic-enhanced continuous flow technology can be utilized to improve quality uniformity and assist in the large-scale production of stimulus-responsive nanomaterials.

The lack of long-term biological safety data is another key factor limiting the clinical application of such materials. Current research focuses mainly on evaluating the safety of stimuli-responsive nanomaterials during wound treatment but lacks in-depth exploration of their metabolic pathways and the toxicity of degradation products, resulting in unclear *in vivo* metabolic kinetic characteristics of the materials and hindering clinical transformation. In the future, comprehensive pharmacokinetic analyses and long-term toxicity assessments of such materials should be performed to improve their safety.

In summary, future research should focus on the precise regulation of material interface properties, the synergistic integration of multistimuli response modules, and the AI-assisted design and screening of stimuli-responsive nanomaterials to overcome existing technical limitations. The advances of these studies will provide theoretical support and technical pathways for developing smart stimuli-responsive nanomaterials tailored to the complex pathological wound microenvironment.

## Figures and Tables

**Table 1:** Summary of application of stimuli-responsive nanomaterials for wound healing

Stimuli-responsive nanomaterials		Components	Role in wound healing	Animal model	Ref.
Endogenous stimuli- responsive nanomaterials	pH-responsive	Allicin@ZIF-8/Ag nanoparticles	Antibacterial, Anti-inflammation, Antioxidant, Angiogenesis	S. aureus-infected full-thickness wound E. coil- infected full thickness wound	49
		Chlorhexidine-Si nanoparticles	Antibacterial	E. coil-infected full-thickness wound	50
	Enzyme- responsive	Ag nanoparticles-TA-COF nanosheets @ ebselen @ polyethylene glycol	Antibacterial, Anti-inflammation	S. aureus-infected full-thickness wound	33
		Linear poly(ethyleneimine)-immobilized nanofiber/ plasmid Human epidermal growth factor	Promote re-epithelization	Full-thickness diabetic wound	51
	ROS- responsive	Trisulfide-derived lipid nanoparticle/ interleukin-4 mRNA	Anti-inflammation, Antioxidant	Full-thickness diabetic wound	52
		Polyvinylpyrrolidone -Ir nanoparticles /Ag nanoparticles / methacrylate gelatin	Antibacterial, Anti-inflammation	Bacteria-infected full-thickness wound	53
	Glucose- responsive	Bacterial cellulose/ polypropylene-Fe@ mesoporous carbon nanosphere/ GOx	Hemostasis, Antibacterial	S. aureus-infected full-thickness wound	54
		Copper nanoclusters/ glucose oxidase / oxidized hyaluronic acid	Reduce blood sugar, Antibacterial	MRSA(Methicillin-Resistant Staphylococcus Aureus)-infected full-thickness diabetic wound	55
	GSH- responsive	CuCo_2_O_4_ nanoflowers	Antibacterial	MRSA-infected full-thickness wound. MRSA-infected full-thickness burn wound	56
	ATP- responsive	Zeolitic imidazolate framework-8/ Indole-3-acetic acid /horseradish peroxidase/ polyacrylamide	Antibacterial	S. aureus-infected full-thickness diabetic wound	57
Exogenous stimuli- responsive nanomaterials	Light- responsive	PDA-PEI-PEG-PMB nanoparticles	Antibacterial	S. aureus-infected full-thickness wound	58
		UCNPs@TiO_2_@GO/ poly(vinylidene) fluoride	Antibacterial, Anti-inflammation	S. aureus-infected full-thickness wound	59
		Au nanoparticle @corn stalk/chitin	Hemostasis, Antioxidant, Antibacterial	Full-thickness wound	60
	Electro- responsive	BaTiO_3_ nanocrystal @MM_Sa_	Antibacterial	S. aureus-infected full-thickness wound	61
		Poly (l-lactic acid) nanofiber	Antibacterial, Promote cell proliferation and migration	Full-thickness wound	62
		Nano-ZnO/ Sodium alginate/ polyvinylidene fluoride	Antibacterial, Promote cell proliferation and migration	Full-thickness wound	63
	Magnetic- responsive	Fe_2_O_3_ nanoparticle labeled MSC-Exo	Promote cell proliferation and migration, Angiogenesis	Full-thickness burn wound	64
		CoFe_2_O_4_ nanoparticle/ polyvinylidene fluoride nanofiber	Antibacterial, Promote the secretion of growth factors	S. aureus-infected full-thickness diabetic wound	65
	Ultrasound- responsive	Platinum nanoparticle assembly/gelatin-methacryloyl	Anti-inflammation	S. aureus-infected full-thickness diabetic wound	66
		(K,Na)NbO_3_ nanocrystals/ reduced graphene oxide/gelatin/polyvin-yl alcohol	Promote angiogenesis and nerve regeneration	S. aureus-infected full-thickness diabetic wound	67
	Thermal- responsive	Polyurethane /polyvinyl butyral	Promote wound contraction	Full-thickness wound	68
		Poly-(lactic acid-co-trimethylene carbonate) nanofibers /methacrylate gelatin /Epinecidin-1@chitosan nanoparticles	Antibacterial, Anti-inflammation	S. aureus-infected full-thickness diabetic wound	69
Dual stimuli- responsive nanomaterials	pH-ROS- responsive	GSC/PBE@Lut (Luteolin)	Antibacterial, Anti-inflammation and Promote angiogenesis	S. aureus-infected full-thickness wound	70
	Light-Electro-responsive	Poly-L-lactic acid-calcium peroxide-reduced graphene oxide	Antibacterial, Promote cell proliferation and migration	E. coil-infected full-thickness wound	71
	Light-Enzyme- responsive	Hyaluronic acid-Porphyrin-based Fe covalent organic frameworks	Antibacterial and Promote angiogenesis	S. aureus-infected full-thickness wound	72

**Table 2:** Light-responsive nanomaterial for wound healing: lighting parameters, photothermal performance, and therapeutic outcomes

	Light-responsive nanomaterial	Lighting parameters	Photothermal conversion efficiency	Surface Temperature	Animal model	Pro-healing effect	Ref.
High temperature photothermal stimulation	CuO@AgO/ZnO nanoparticle	808 nm laser, 2 W/cm^2^, 7min	23%	55 °C	S. aureus-infected full-thickness wound	Antibacterial	147
	Highly graphitic- N-doped GQDs	1064 nm laser, 1 W/cm^2^, 5 min	50.4%	50.11 °C	MRSA-infected full-thickness wound	Antibacterial	148
	Fe_3_O_4_ nanoparticle	808 nm laser, 1 W/cm^2^, 10 min	28.5%	50.5 °C	S. aureus-infected full-thickness wound	Antibacterial	149
	Polypropylene/ polyacrylonitrile _x%polydopamine_	808 nm laser, 0.2 W/cm^2^, 3 min	-	60 °C	S. aureus-infected full-thickness diabetic wound	Antibacterial, Promote volatilization of the exudate	150
	MoS_2_/Silk Sericin	808 nm laser, 1 W/cm^2^, 5 min	-	> 50 °C	MRSA-infected full-thickness wound	Antibacterial	151
Mild temperature photothermal stimulation	mPDA@deferoxamine@chitosan-graft-third generation poly(amidoamine) polymer with terminal S-nitrosothiol groups	808 nm laser, 2.5 W/cm^2^, 10 min	32.3%	45 °C	S. aureus-infected full-thickness wound	Synergistic antibacterial, Anti-inflammation, Angiogenesis	30
	Curcumin-based metal- organic framework	808 nm laser, 1.5 W/cm^2^, 5 min	-	43.9 °C	S. aureus-infected full-thickness wound	Synergistic antibacterial, Anti-inflammation, Angiogenesis, Promote nerve regeneration	152
	CuSi nanowires	808 nm laser, 1 W/cm^2^, 15 min	-	45 °C	S. aureus-infected full-thickness wound	Synergistic antibacterial, Angiogenesis	153
	CuS@BSA nanoparticle	980 nm laser, 0.8 W/cm^2^, 4 min	42%	42 °C	Full-thickness wound	Inducing MSCs differentiation to fibroblasts	140
	Poly-l-lactic acid/quaternized chitosan/black phosphorus/Hemoglobin	808 nm laser, 1.5 W/cm^2^, 3 min	-	40 °C	S. aureus-infected full-thickness diabetic wound	Synergistic antibacterial, Angiogenesis	154

## Data Availability

Data will be made available on request.

## Abbreviations

ROS: Reactive oxygen species
MMPs: Matrix metalloproteinases
MRSA: Methicillin-Resistant Staphylococcus Aureus
Ag NPs: Silver nanoparticles
PST: Polyserotonin
PHMB: Polyhexamethylene biguanide
α-MnS NPs: α-phase manganese sulfide nanoparticles
OXD: Oxidase
^1^O_2_: Singlet oxygen
SOD: Superoxide dismutase
CAT: Catalase
SiNPs: Silica nanoparticles
CHX: Chlorhexidine
GOx: Glucose oxidase
O_2_·⁻: Superoxide anions
OH: Hydroxyl radicals
COFs: Covalent organic frameworks
EBS: Ebselen
SplB: Serine protease-like B enzyme proteins
hEGF: Human epidermal growth factor
HA: Hyaluronic acid
HAnase: Hyaluronidase
Pt NPs: Platinum nanoparticles
TEM: Transmission electron microscope
GSH: Glutathione
ATP: Adenosine triphosphate
GPx: Glutathione peroxidase
N-CDs: Nitro-quantum dots
IAA: Indole-3-acetic acid
PTDT: Photothermal-photodynamic synergistic effects
MCC/CS NPs: Monocarboxylic phenol/chitosan nanoparticles
PDA@ZnO NPs: Polydopamine-coated zinc oxide nanoparticles
QT: Quercetin
QCS: Quaternary ammonium chitosan
CP NPs: Cu_x_O@PDA nanoparticles
HIF-1α: Hypoxia inducible factor-1α
HUVECS: Human umbilical vein endothelial cells
MSCs: Mesenchymal stem cells
BPA: Bisphenol A
TCSN: Temperature controlled smart nanoparticle
UCNPs: Upconversion nanoparticles
CCPI NPs: CaSiO_3_-ClO_2_ @PDA-ICG nanoparticles
TGF-β1: Transforming Growth Factor Beta 1
EMT: Epithelial-mesenchymal transition
NOS: Nitric oxide synthase
ZPFSA: ZnO nanoparticle-modified polyvinylidene fluoride/sodium alginate piezoelectric hydrogel
c-MWCNTs-VAN: Carboxylated carbon nanotubes/vancomycin hydrochloride
PVDF: Polyvinylidene fluoride
PEO: Polyethylene oxide
BT: Barium titanate
BTO@MMSa: Barium titanate@macrophage membranes pre-activated by Staphylococcus aureus
PTEN: Phosphatase and tensin homolog
PAMPs: Pathogen-associated molecular patterns
TENGs: Triboelectric nanogenerators
PPY/PCL: Polypyrrole/Polycaprolactone
PLGA: Poly(lactic-co-glycolic acid)
EGD: Electro-Generating Dressing
PNA: Platinum nanoparticle assemblies
OV-TiO_2_/CuO_2_: Oxygen-rich vacancy porous titanium dioxide nanosheets/ultrafine copper peroxide nanoclusters
PU/PVB: Polyurethane/polyvinyl butyral
CGH: Copper nanoclusters/Gox/oxidized hyaluronic acid
CuNCs: Copper nanoclusters
PLLA: Poly-L-lactic acid
rGO: Reduced graphene oxide
CaO_2_: Calcium peroxide
Hsp 90: Heat shock protein 90
PDA: Polydopamine
C@M@P: Ceria-molybdenum disulfide nanoparticles
CDs: Carbon quantum dots
